# Characterization of the pathogenicity and mechanisms underlying the pathogenesis of *Apibacter raozihei*, a potential bacterial pathogen

**DOI:** 10.1080/21505594.2025.2586201

**Published:** 2025-11-22

**Authors:** Yuanmeihui Tao, Suping Zhang, Kexin Qi, Wenbo Luo, Sihui Zhang, Jing Yang, Dong Jin, Shan Lu, Yuyuan Huang, Han Zheng, Jianguo Xu

**Affiliations:** a National Key Laboratory of Intelligent Tracking and Forecasting for Infectious Diseases, National Institute for Communicable Disease Control and Prevention, Chinese Center for Disease Control and Prevention, Beijing, China; b Institute of Parasitic and Endemic Disease Prevention and Control, Sichuan Center for Disease Control and Prevention, Chengdu, Sichuan, PR China; c Department of Epidemiology and Biostatistics, School of Public Health, Peking University, Beijing, China; d Research Units of Discovery of Unknown Bacteria and Function, Chinese Academy of Medical Sciences, Beijing, China; e Guangxi Colleges and Universities Key Laboratory of Prevention and Control of Highly Prevalent Diseases, School of Public Health, Guangxi Medical University, Nanning, Guangxi, China; f Research Center for Reverse Microbial Etiology, Workstation of Academician, Shanxi Medical University, Taiyuan, China; g Key Laboratory of Coal Environmental Pathogenicity and Prevention, Shanxi Medical University, Taiyuan, China

**Keywords:** *Apibacter raozihei*, potential pathogen, inflammatory response, comparative transcriptome analysis, pathogenicity evaluation

## Abstract

*Apibacter raozihei* is a Gram-negative bacterium isolated from the feces of bats, and there is a scarcity of information regarding its genomic and pathogenicity characteristics. This study systematically evaluated the pathogenic potential of *A*. *raozihei* by comparing the survival rates, bacterial loads in the peripheral blood and organs, histopathological lesions, and the production of proinflammatory cytokines in C57BL/6 mice infected with strains HY037 and HY041^T^ of *A*. *raozihei*. The findings revealed that mice infected with *A*. *raozihei* had low survival rates, with significant differences between the HY041^T^ and HY037 strains. The mice infected with HY041^T^ exhibited more severe pulmonary histopathological damage and higher levels of proinflammatory cytokines compared to those infected with HY037. These results demonstrated that *A*. *raozihei* is lethal in mice, causing significant pulmonary histopathological damage accompanied by the dysregulated secretion of proinflammatory cytokines in lung tissues and blood. The molecular mechanisms underlying their virulence were investigated through comparative analyses of the genomic and transcriptional profiles of HY041^T^ and HY037. The findings revealed that the genes encoding outer membrane protein A (OmpA), three peptidases, heat shock proteins, and proteins involved in lipopolysaccharide biosynthesis, and iron acquisition represent potential virulence factors of *A*. *raozihei*. In conclusion, this study established *A*. *raozihei* as a potential pathogen and identified significant differences in virulence across its strains, thereby enhancing our understanding of the pathogenesis of *A*. *raozihei*.

## Introduction

*Apibacter raozihei* is a Gram-negative, rod-shaped, nonmotile, facultatively anaerobic bacterium isolated from the feces of bats. It belongs to the genus *Apibacter* within the family *Weeksellaceae*, order *Flavobacteriales*, and class *Flavobacteriia Flavobacteriiaea* (https://www.namesforlife.com/). The genus *Apibacter* consists of four species, namely, *A*. *adventoris* [1], *A*. *mensalis* [2], *A*. *musca*e [3], and *A*. *raozihei* [4]. *A*. *adventoris* was discovered in 2016 and represents the first species identified in the genus. *A*. spp. have been isolated from honeybees, house flies, and rove beetle, *A*. *adventoris* constitutes approximately 35% of all the microbial communities in rove beetle [5]. Current research has primarily focused on honeybee microbiomes rather than the characterization of individual species, and there is a scarcity of information regarding the pathogenicity of the *Apibacter* genus. However, several species in the genus *Chryseobacterium*, within the *Weeksellaceae* family, including *C*. *indologenes* [6,7], *C*. *gleum* [8], *C*. *meningosepticum* [9], and *C*. *arthrosphaerae* [10], have been shown to exhibit pathogenicity. Similarly, several species in the *Elizabethkingia* genus, including *El*. *miricola* [11], *El*. *anophelis* [12], and *El*. *meningoseptica* [13], have been reported to cause various infectious diseases. Notably, *El*. *anophelis* represents the most prevalent pathogen within the genus and has been responsible for causing multiple outbreaks in France [12], Singapore [14], Hong Kong [15], Taiwan [16], and the United States [17]. These pathogens predominantly affect newborns, immunocompromised individuals, and hospitalized patients, causing various conditions, including bacteremia [18], pneumonia [19], meningitis [13], and sepsis [20].

*A*. *raozihei* was recently isolated and identified in our previous study; however, there is a scarcity of information pertaining to its significance in public health. Therefore, understanding the mechanism underlying the pathogenesis of *A*. *raozihei* is essential for assessing its potential public health risks and for developing effective therapeutic strategies. The present study evaluated the virulence of various *A*. *raozihei* strains in C57BL/6 mice through survival assays. The pathogenic characteristics of the strains were elucidated by comparative analyses of the bacterial loads, histopathological alterations, and inflammatory responses in C57BL/6 mice infected with the highly pathogenic HY041^T^ strain and the less pathogenic HY037 strain of *A*. *raozihei*. The mechanism underlying the pathogenesis of *A*. *raozihei* was further investigated by comparative analyses of the genome-wide transcriptional profiles of the highly pathogenic HY041^T^ strain following their interactions with A549 cells, with those of the less pathogenic HY037 strain.

## Materials and methods

### Experimental infection

#### Survival assay

C57BL/6 mice (6 weeks old, female) were purchased (Sibeifu) and injected intraperitoneally with 5 × 10^8^ colony-forming units (CFU) of the *A. raozihei* strains in 1 ml PBS or 1 ml of only PBS as the control group. Each group contained ten mice in one cage; a total of 90 mice were randomly assigned to cages, each cage was randomly assigned to different infection groups of *A. raozihei* strains and a control group in this experiment. The death event that occurred to the mouse was recorded as 1; the mouse survival record is 0.

The sample size was determined based on the methods in the literature [21]. After the injection, the mice were placed in the same environment (Biosafety level 2) for feeding. During the experiment, each group of mice was randomly grabbed and tested in the cage in each assay. For each mouse, two different researchers were involved as follows: the first researcher performed the experiment, this researcher was the only one who knew the experimental treatment group assignment. The second researcher was responsible for the results analysis and processing.

#### Comparison of cytokine production in the serum, bacterial loads, histopathological lesions in different organs, and inflammatory mediators in the lungs of mice infected with A. raozihei strains HY037 and HY041^T^

C57BL/6 mice (6 weeks old, female) were injected intraperitoneally with 1 × 10^8^ CFU of the strains HY037, HY041^T^ or 1 ml PBS, respectively. Each group contained five mice in one cage, a total of 30 mice were randomly assigned to each group used in this experiment. The peripheral blood of each infected mouse was collected at 12 and 24 h post-infection. The cytokines levels in the serum of infected mice were determined by ELISA kits (R&D SYSTEMS, PMTA00B, and PM6000B) according to the manufacturer’s instructions. The brain, liver, spleen, kidney, intestine, and lung of mice were collected. Half of them were fixed in 4% paraformaldehyde, dehydrated, and embedded in paraffin. The organs were cut into 4 mm tissue sections. The tissue sections were stained with hematoxylin-eosin and evaluated by the post-examination blinding method [22].

One hundred microliters of serial tenfold dilutions of peripheral blood of each infected mouse were plated onto the THB agar plates. Another half of the organ samples were accurately weighed thoroughly and ground to homogenate and placed in 1 ml of PBS. One hundred microliters of serial tenfold dilutions of organ homogenate were plated onto THB agar plates.

Lung samples were treated using the QiAzol lysis Reagent (Qiagen, 79,306) with a steel bead in the tube and homogenized by Tissueputor 2. Total RNA was extracted and purified by a RNeasy Plus Universal Minikit (Qiagen, 73,404). The quality and quantity of total RNA were evaluated by a nanodrop 1000 instrument. The transcription level of cytokines (IL-6 and TNF-α) in the lungs was determined by qRT-PCR. cDNA library was constructed by incubation with a Prime Script RT reagent kit (TAKARA RR037B) at 37°C for 15 min, reverse transcriptase was inactivated with heat treatment at 85°C for 5 s and the samples were stored at 4°C. TB Green® Premix Ex Taq TM II (TAKARA, RR820) was used in qPCR according to the manufacturer’s protocol.

Beta-2-microglobulin (β_2_M) is a protein-coding gene that is found in almost all nucleated cells and was used as a normalizing gene in this study. The quantitation of the differences between groups was calculated using the 2^−ΔΔCt^ method. The transcription values were expressed as mean ± standard deviation. These Primers used in the present study are listed in Table S1. All the survival and experimental mice were sacrificed by cervical dislocation, and the procedures accordance with applicable veterinary guidelines, such as the American Veterinary Medical Association.

### Comparative transcriptome analysis

#### A549 cell treatment

3 × 10^5^ A549 cells per well were plated into 24-well flat-bottomed plates (Corning, 3524) and maintained in 5% CO_2_ 37°C for 24 h to allow the cells to grow to confluency (~1.0 × 10^6^ cells per well, 48 wells totally) before treatment with *A. raozihei*. The medium was changed every 24 hours. The bacterial suspensions of the two *A. raozihei* strains were prepared in F12/DMEM medium containing 10% FBS to obtain a desired concentration of 5 × 10^7^ CFU/ml for each strain. A549 cells were infected with the prepared bacterial suspensions of the two *A. raozihei* strains for 8 and 16 hours. The supernatants were removed, and the bacteria were harvested by repeated gentle washing with PBS. The washing solution was filtered by a filter (5 μm) to remove the cell debris, and the precipitate of bacteria was harvested by centrifuging at 4000 r/min. At least 1 × 10^9^ CFU bacteria were used for further RNA extraction in each group.

#### High-throughput sequencing

Following the manufacturer’s recommendations, total RNA from the pallet was extracted using TRIzol (Thermo Scientific, 15,596,026). The MICROB Express Kit (Ambion, AM1901) and MICROB Enrich Kit (Ambion, AM1905) were used to enrich bacterial mRNAs in total RNA samples by eliminating rRNAs and polyadenylated mRNAs. All RNA samples were quantified and checked for protein and reagent contamination by Nanodrop ND1000 spectrophotometer. The integrity of RNA was detected by running the RNA sample on an Agilent Bioanalyzer RNA 6000 Nano/Pico Chip. At least 100 ng purified total RNA samples with RNA Integrity Number values greater than seven were applied to construct RNA-Seq libraries.

Illumina TruSeq RNA libraries were prepared using the NEBNext Ultra RNA Library Prep Kit (Illumina, E7530). The cDNA library quality was assessed on an Agilent Bioanalyzer DNA high-sensitivity chip. The constructed cDNA libraries with fragment lengths of approximately 300 bp were sequenced on an Illumina X-TEN platform. The volume and value of RNA-seq raw data are shown in Table S2.

#### RNA-seq data analysis

The reads were filtered by fastp (Version 0.23.2) by removing the low-quality reads, poly-N-terminal reads, and reads with adaptors. After filtration, the reads were mapped to the HY041^T^ and HY037 genomes by HISAT2 (Version 2.0). The homologous genes were determined by the website service Orthovenn3 (https://orthovenn3.bioinfotoolkits.net/). The number of reads mapped on the HY041^T^ genome was counted by StringTie (Version 2.2.1). All settings were the default settings unless otherwise stated. Differential gene expression was calculated by the R package DESeq2. The difference was considered significant when log_2_FoldChange ≥1.5 and adjusted *p-value* < 0.05. The annotation of differentially expressed genes was predicted by the Evolutionary Genealogy of Genes: Nonsupervised Orthologous Groups Database (eggnog, Version 5.0). The prediction of effectors secreted by the Type IX secretion system (T9SS) was the NCBI website service CD-search (https://www.ncbi.nlm.nih.gov/Structure/cdd/wrpsb.cgi).

#### Nucleotide sequence accession number

The raw transcriptome sequence data have been deposited in the National Center for Biotechnology Information (NCBI) Sequence Read Archive (https://www.ncbi. nlm.nih.gov/sra) under accession numbers SRR22696154 ~ SRR22696156, SRR22696158 ~ SRR22696163, SRR22699715 ~ SRR22699724.

### Statistics

Survival analysis was performed by the log-rank (Mantel-Cox) test. Differences in cytokine levels (TNF-α and IL-6), and bacterial loads between infected and control mice were calculated by the Multiple-comparison corrections (Bonferroni). *p < 0.05 was considered significant. All statistical analyses were performed using GraphPad Prism 8.

## Results

### Survival analysis of mice infected with A. raozihei

To investigate the pathogenicity of *A*. *raozihei*, we assessed the survival rates of C57BL/6 mice infected with eight strains of *A*. *raozihei*, namely, HY037, HY039, HY041^T^, HY101, HY113, HY204, HY281, and HY292. The infected mice displayed various clinical manifestations, including a reduction in activity, tremors, ocular discharge, and increased mortality.

**Figure 1.**
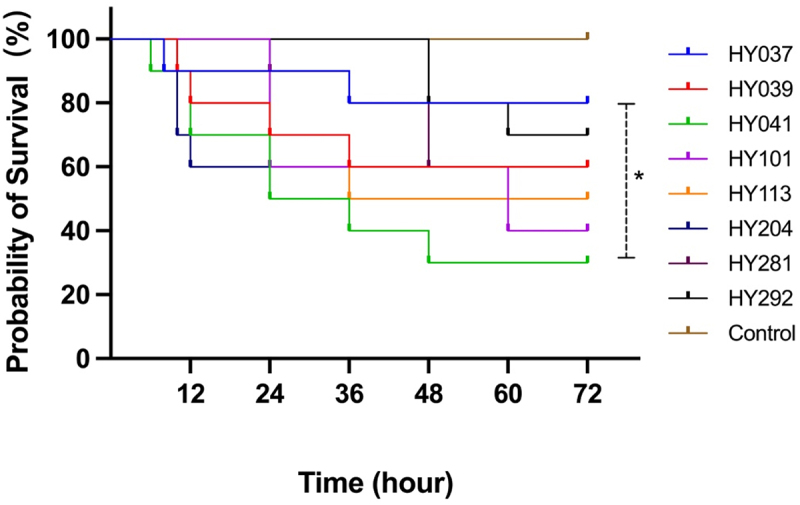
Survival analysis of C57BL/6 mice following infection with different *A*. *raozihei* strains. The mice were injected with the HY037, HY039, HY041^T^, HY101, HY113, HY204, and HY292 strains of *A*. *raozihei* (*n* = 10), at a dose of 5 × 10^8^ CFU/mL per mouse. Survival was monitored over a 72-hour period. Statistical significance was assessed using the log-rank (Mantel-Cox) test; *p < 0.0332.

The survival rates of mice infected with HY037, HY039, HY041^T^, HY101, HY113, HY204, HY281, and HY292 at a dose of 5 × 10^8^ CFU/mL were 80%, 60%, 30%, 40%, 50%, 60%, 60%, and 70%, respectively, 72 hours post-infection (hpi) (Figure 1). The majority of fatalities occurred within 24 hpi, of which the mice infected with the HY041^T^ strain exhibited the lowest survival rate of 30%, indicating the hypervirulence of this strain. Statistical analysis using the log-rank (Mantel-Cox) test revealed significant differences between the survival curves for the HY037 and HY041^T^ strains, corresponding to the highest and lowest survival rates, respectively. These findings demonstrated that *A*. *raozihei* infections are lethal in murine models, and that there are strain-dependent variations in the degree of virulence.

### Organ pathogenicity of the HY041^T^ and HY037 strains of *A. raozihei*

The pathogenic potential of *A*. *raozihei* was characterized by analyzing bacterial colonization and histopathological changes in various organs of mice infected with the highly virulent HY041^T^ strain. Although bacteria were isolated from the brain, lungs, liver, spleen, and kidneys, significant histopathological lesions, characterized by hemorrhage and the infiltration of lymphocytes and neutrophils, were only observed in the lungs within 48 hpi (Fig. S1).

**Figure 2.**
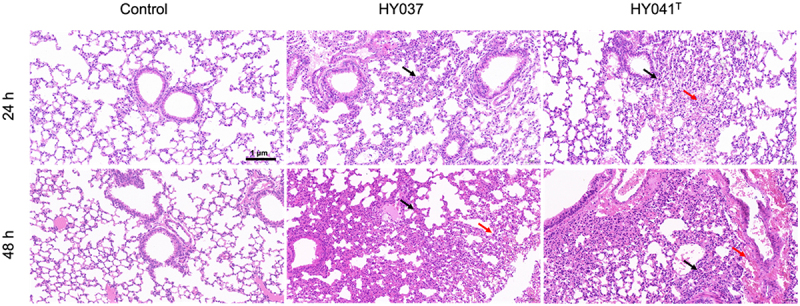
Pulmonary inflammatory response in C57BL/6 mice infected with *A*. *raozihei*. C57BL/6 mice were intraperitoneally injected with the HY037 and HY041^T^ strains at a dose of 10^8^ CFU/mL per mouse. The lung tissues were subjected to hematoxylin-eosin staining for histopathological examination. The black arrows denote the infiltration of lymphocytes and neutrophils, whereas the red arrows indicate areas of hemorrhage (*n* = 5). The images were captured using a microscope at a magnification of 20×; scale bars: 1 µm.

Changes in the lung tissues of mice infected with the HY037 or HY041^T^ strains at 24 and 48 hpi revealed that both the strains induced the infiltration of lymphocytes and neutrophils into the vascular space and the thickening of alveolar septa at 24 hpi. However, the HY037 strain induced mild inflammatory infiltration, whereas the HY041^T^ strain triggered moderate inflammatory infiltration at 24 hpi. Notably, pulmonary hemorrhage was observed exclusively in mice infected with the HY041^T^ strain at 24 hpi (4/5 mice; 80%), whereas mice infected with the HY037 strain developed hemorrhage at 48 hpi (Figure 2). The inflammatory response intensified in both the groups by 48 hpi. These observations indicated that *A*. *raozihei* induced progressive pulmonary inflammation in mice; however, the pathogenicity of the HY041^T^ strain was significantly higher than that of the HY037 strain, as evidenced by the earlier onset of hemorrhage and the enhanced infiltration of leukocytes.

### Induction of inflammatory responses induced by the HY041^T^ and HY037 strains of *A. raozihei* in infected mice

The differential pathogenicity between the HY037 and HY041^T^ strains was further examined by comparative analyses of the serum concentrations and transcription levels of proinflammatory cytokines in the lung tissues of mice infected with both the strains.

**Figure 3.**
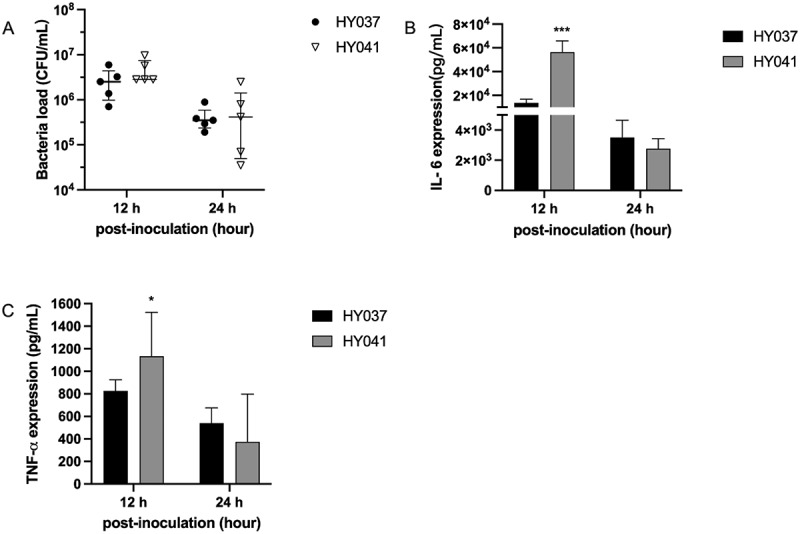
Expression of cytokines and bacterial loads in the peripheral blood of C57BL/6 mice infected with *A*. *raozihei*. Serum expression levels of (A): IL-6 and (B): TNF-α. (C): bacterial load in peripheral blood. The mice were infected with the HY041^T^ and HY037 strains of *A*. *raozihei* at a dose of 10^8^ CFU/mL per mouse (*n* = 5). The data are presented as the mean ± standard deviation (sd); ***p < 0.001 and *p < 0.05.

The serum concentrations of the cytokines, Interleukin 6 (IL-6) and Tumor Necrosis Factor Alpha (TNF-α), were highest in mice infected with both HY037 and HY041^T^ at 12 hpi (Figure 3(A,B)). Meanwhile, the serum levels of IL-6 and TNF-α were significantly higher in mice infected with the HY041^T^ strain compared to those in mice infected with the HY037 strain at 12 hpi. Despite the differences in the levels of these cytokines, the bacterial load in the peripheral blood remained comparable between mice infected with HY037 or HY041^T^, suggesting that the variations in cytokine production were not attributable to differences in bacterial load (Figure 3(C)).

**Figure 4.**
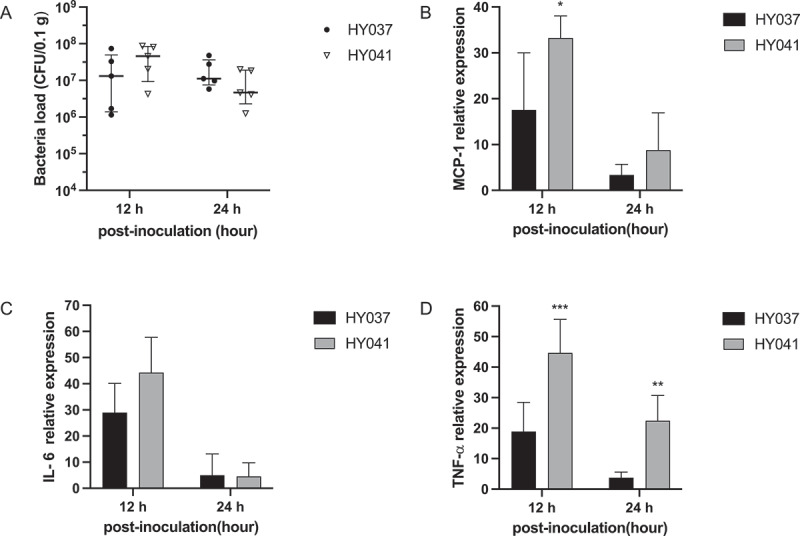
Relative expression levels of inflammatory factors and bacteria loads in the lung tissues of C57BL/6 mice infected with *A*. *raozihei*. (A): bacterial load in the lung tissues of infected mice. Relative expression levels of (B): MCP-1, (C): IL-6, and (D): TNF-α in the lung tissues of mice infected with the HY041^T^ and HY037 strains of *A*. *raozihei*. The data are presented as the mean ± standard deviation (SD); *n* = 5; **p < 0.01 and ***p < 0.001.

Analysis of the pulmonary transcriptional levels of Monocyte chemoattractant protein-1 (MCP-1), IL-6, and TNF-α revealed that their expression was highest at 12 hpi following infection with both the HY037 and HY041^T^ strains (Figure 4). The transcriptional levels of MCP-1 and TNF-α were significantly elevated in mice infected with the HY041^T^ strain at 12 hpi (Figure 4(B,D)), while those of IL-6 were comparable between the groups infected with HY037 or HY041^T^ (Figure 4(C)). Also, the comparable bacterial load suggesting that the variations in cytokine production in the lung tissues were not attributable to differences in bacterial load. These findings, in conjunction with the observed cytokine and chemokine expression patterns, indicated that *A. raozihei* can trigger inflammatory responses at the early phase of infection, and the HY041^T^ strain significantly enhanced the potential to trigger inflammatory responses compared to the HY037 strain.

### Differential expression of virulence-related genes of *A. raozihei* following interactions with A549 cells

The gene transcription patterns were analyzed following interactions with A549 cells to identify the virulence factors corresponding to the different strains of *A*. *raozihei*. The results of transcription sequencing yielded > 10^7^ raw reads, with a Q20 percentage > 90%, and over 10^6^ mRNA reads. The results of genome alignment satisfied the necessary criteria for subsequent analyses (Table S2).

The differentially expressed genes (DEGs) between the two strains following interactions with A549 cells were subsequently identified. A total of 122 and 22 DEGs were identified from HY041^T^ and HY037, respectively, of which 44 and 9 were upregulated, respectively, at 8 hpi. The responses increased substantially by 16 hpi, and 619 and 747 DEGs were identified from HY041^T^ and HY037, respectively, of which 285 and 383 were upregulated, respectively (Fig. S3). Due to the limited functional annotation of *A*. *raozihei* genes, the DEGs from the interacting groups did not form significant functional clusters, as revealed by analysis with the DAVID web server. To address this limitation, the information obtained by predicting the annotations with eggNOG-mapper was incorporated in the analysis. We also performed virulence factor prediction based on the complete genomic sequence of *A. raozihei* strains. Furthermore, the reported virulence factors within the *Weeksellaceae* family (Table S3 and S4) was systematically summarized based on NCBI databases, followed by comparative genomic analysis against *A. raozihei* sequences. Three aforementioned approaches were used to functional gene annotation.

The homologous genes that were upregulated in both the HY037 and HY041^T^ strains were identified as the potential virulence factors of *A*. *raozihei*. A total of 2,873 homologous genes were subsequently identified between the HY037 and HY041^T^ strains (Fig. S2). Four strain-specific clusters were identified in the HY041^T^ strain, whereas only one was found in HY037 (Table S5). Functional annotation of the genes specific to HY041^T^ revealed that the *HY041_GM000346*/*HY041_GM001761* gene cluster is homologous to the *SprB* gene of *Flavobacterium johnsoniae* UW101 [23], which encodes a component of the Type IX secretion system (T9SS). The remaining strain-specific genes encoded hypothetical proteins with undefined functions.

Three and 165 homologous genes were found to upregulated in both the HY037 and HY041^T^ at 8 and 16 hpi, respectively (Table S6). Among these upregulated homologous genes, the expression of lipopolysaccharide (LPS) kinase, encoded by *HY041_GM000438*, is a crucial enzyme involved in the phosphorylation of the LPS core, and was found to be upregulated in both the strains after 8 hours of interactions with A549 cells [24] (Table 1). Following 16 hours of interaction, the genes encoding the outer membrane protein A (OmpA), the type 8 capsule J (Cap8J) enzyme that partakes in the synthesis of the capsular polysaccharide, and three peptidases, namely, AlgW, ClpP, and peptidase M48, were upregulated and demonstrated high similarities with the virulence factors from pathogens within the *Weeksellaceae* family, which led to their identification as the virulence-related genes of *A*. *raozihei* (Table 1 , Table S3). The genes encoding proteins involved in the iron acquisition systems were also significantly upregulated in both the strains, including the TonB-dependent receptor, Fur proteins, and the Fe^3+^-siderophore ABC transporter permease. These components were found to be homologous to the known virulence factors of *Riemerella anatipestifer*, a pathogenic bacterium in the *Weeksellaceae* family [25] (Table 1 , Table S3). Furthermore, the *groEL*, *clpP*, and *dnaK* genes, which encode heat shock proteins (HSPs), were upregulated in both the HY037 and HY041^T^ strains of *A*. *raozihei*, indicating that they potentially function as defensive virulence factors during host interaction.

**Table 1.** Upregulated genes associated with the virulence of HY041^T^ and HY037 strains, as determined by RNA-seq following interactions with A549 cells. The DEGs were analyzed using DESeq2, with a log_2_(fold change) cutoff of ±1.5 (*n* = 3). The groups that were not treated with A549 cells served as the controls for each strain.

Related pathways	Gene name	Predicted function (gene)	fold change (log_2_)	
	8 h interaction		HY041^T^	HY037
LPS biosythesis	HY041_GM000438	Lipopolysaccharide kinase (Kdo WaaP) family	2.82	3.90
	16 h interaction			
Capsular biosythesis	HY041_GM000363	cap8J capsular polysaccharide synthesis enzyme Cap8J	2.41	2.26
Outer membrane protein	HY041_GM000853	Out membrane protein A(*ompA*)	1.77	1.77
Peptidase	HY041_GM002298	algW peptidase (*algW*)	3.15	2.95
	HY041_GM002093	ATP dependent Clp protease proteolytic subunit (*clpP*)	2.41	2.81
	HY041_GM002489	Peptidase M48	1.74	2.21
Iron acquisition	HY041_GM000579	TonB-dependent receptor	5.67	4.98
	HY041_GM000653	Ferric uptake regulator (Fur) proteins (*fur*)	1.85	3.10
	HY041_GM001766	Ferric uptake regulator (Fur) proteins (fur)	1.76	1.67
	HY041_GM002098	Fe3±siderophore ABC transporter permease	7.73	1.96
	HY041_GM002232	TonB-dependent receptor	2.60	2.53
Heat shock related proteins	HY041_GM000957	heat shock protein 60 family chaperone GroEL(*groL*)	5.30	4.89
	HY041_GM000958	molecular chaperone GroES(*groS*)	4.30	4.42
	HY041_GM002483	Hsp90 protein(*htpG*)	4.79	4.93
	HY041_GM002495	molecular chaperone DnaJ(*dnaJ*)	5.45	5.17
	HY041_GM002496	molecular chaperone GrpE(*grpE*)	2.95	3.52
	HY041_GM002895	Heat shock 70 kDa protein(*dnaK*)	5.14	4.78

The potential virulence determinants contributing to the hypervirulence of HY041^T^ were identified by comparing its transcriptional profile with that of the HY037 strain during interactions with A549 cells. The findings revealed that 231 and 158 genes were differentially expressed in the two strains at 8 and 16 hpi, respectively. The transcriptional levels of 202 and 143 genes in the group interacted with the HY041^T^ strain were higher than those of the corresponding homologous genes in the HY037 strain at 8 and 16 hpi, respectively (Table S7). Among the genes with elevated expression levels following treatment with HY041^T^, 2 and 18 genes were upregulated at 8 and 16 hours, respectively, compared to those at 0 hours, as detailed in Table 2. The *lptB* gene, which encodes an ATP-binding protein in the LPS export system and is implicated in the transport of LPS from the inner membrane to the outer membrane of *Escherichia coli* [26], was upregulated by 45.8-fold in the HY041^T^ strain following interactions with A549 cells at 8 hpi. Furthermore, the expression of the *lptB* gene in the HY041^T^ strain was 22.2-fold higher than that in the HY037 strain (Table 2), suggesting the increased transportation of LPS to the outer membrane. The gene clusters involved in glycerol metabolism were upregulated in both the strains at 16 hpi (Table 1). Notably, the expression of *glpT*, which is involved in the transportation of glycerol-3-phosphate, upregulated 55.7-fold after interaction with cells for 16 hours, and was expressed 17.2 higher in the HY041^T^ strain than that in HY037, indicating an increase in the transportation of glycerol-3-phosphate (Table 2) [27]. Additionally, the *BshB1* gene, which encodes an enzyme that catalyzes the second step in the biosynthesis of bacillithiol [28–30], was upregulated 4.9-fold in the HY041^T^ strain after interaction with cells for 16 hours, and 8.5-fold higher expressed than that in HY037 (Table 2), suggesting an increased production of bacillithiol in the HY041^T^ strain. Furthermore, the expression levels of 27 genes encoding RHS repeat-associated core domain proteins as well as members of the RHS family were higher in the HY041^T^ strain than in the HY037 strain at 8 and 16 hpi. Three of these genes, namely, *HY041_GM000182*, *HY041_GM000243*, and *HY041_GM001990*, were identified as encoding T9SS effector proteins (Table S8 , S9).

**Table 2.** Comparative analysis of upregulated virulence genes in HY041^T^ relative to those in HY037 and the respective control groups following interactions with A549 cells. The DEGs were analyzed using DESeq2, with a log_2_(fold change) cutoff of ±1.5 (*n* = 3).

	Gene locus	Fold change	Product	Preferred name
8 h	HY041_GM001599	22.23	lipopolysaccharide export system ATP-binding protein	*lptB*
	HY041_GM002139	1555.32	hypothetical protein	N/A
16 h	HY041_GM000063	72.82	hypothetical protein	N/A
	HY041_GM000236	35.73	hypothetical protein	N/A
	HY041_GM000241	1202.39	protein of unknown function (DUF4279)	N/A
	HY041_GM000288	131.7	hypothetical protein	N/A
	HY041_GM000289	119.65	RHS repeat-associated core domain-containing protein	N/A
	HY041_GM000290	227.63	hypothetical protein	N/A
	HY041_GM000535	295.68	hypothetical protein	N/A
	HY041_GM000693	8.67	SAM-dependent methyltransferase	N/A
	HY041_GM000698	110.38	hypothetical protein	N/A
	HY041_GM000867	5342.18	hypothetical protein	*hdc*
	HY041_GM000920	17.02	glycerol-3-phosphate transporter	*glpT*
	HY041_GM001200	28.88	Carbonic anhydrase	N/A
	HY041_GM001223	4.39	exosortase family protein XrtF	*groEL*
	HY041_GM001318	4.71	tRNA-splicing ligase RtcB	*rtcB*
	HY041_GM001419	4.63	hypothetical protein	CP_0628
	HY041_GM001682	8.46	bacillithiol biosynthesis deacetylase BshB1	*bshB1*
	HY041_GM002014	67.91	hypothetical protein	N/A
	HY041_GM002803	4	protease	*pfpI*

Altogether, the findings revealed that the proteins involved in the biosynthesis and transport of LPS, iron acquisition, and the heat shock response, as well as OmpA, Cap8J, and three peptidases (AlgW, ClpP, and peptidase M48), represented the potential virulence factors of *A*. *raozihei*.

## Discussion

### Pulmonary lesions and cytokine overexpression during the early phase of infection caused high mortality in infected mice

To understand the mechanisms underlying the mortality of mice infected with *A*. *raozihei*, previous clinical cases investigating pathogens in the *Weeksellaceae* family, including *C*. *indologenes*, *El*. *meningoseptic*, *El*. *anophelis*, *Weeksella virosa*, and *Empedobacter brevis*, were comprehensively analyzed. These pathogens are responsible for causing severe neonatal meningitis, nosocomial pneumonia, and bacteremia, and contribute to the high mortality observed in neonates and immunocompromised individuals [31]. The mortality rates can reach up to 70% following infection with *El*. *anophelis* [15], which align with the results of survival analysis using mice infected with *A*. *raozihei* in this study. The clinical reports demonstrated that *El*. *anophelis* infections manifest as bacteremia (59.7% of isolates from blood), respiratory tract infections (11.9%), and catheter-related infections (10.4%) [32]. The most common diagnoses of *El*. *anophelis* infections are pneumonia (*n* = 5), catheter-related bacteremia (*n* = 4), neonatal meningitis (*n* = 3), nosocomial bacteremia (*n* = 2), and neutropenic fever (*n* = 1) [15]. Similar to those of *El*. *anophelis*, the primary clinical manifestations of *A*. *raozihei* are bacteremia and pneumonia. A substantial load of *A*. *raozihei* was isolated from the peripheral blood and lung tissues of infected mice, and significant lesions were observed in the lung tissues. These predominant clinical manifestations were found to be consistent with those of pathogenic bacteria within the *Weeksellaceae* family.

The inflammatory factors related to the pathogenic mechanisms were investigated in this study to identify the factors underlying the mortality of mice infected with *A*. *raozihei*, particularly the HY041^T^ strain. Previous studies have demonstrated that the dysregulation of cytokine secretion plays a significant role in early mortality during Gram-negative infections [33]. Consistent with these findings, the majority of mice deaths occurred within 24 hours in this study, and the production of cytokines was elevated in the blood and lung tissues of mice infected with *A*. *raozihei* in the early phase of infection. The excessive production of cytokines contributed to the early mortality of mice infected with *A*. *raozihei*. Comparison of the pathogenicity of the HY041^T^ and HY037 strains revealed significantly higher mortality rates, more severe pulmonary lesions, and elevated expression levels of inflammatory cytokines and chemokines (including MCP-1) in mice infected with the HY041^T^ strain. MCP-1, a key chemokine involved in the recruitment of monocytes/macrophages, influenced the severity of organ lesions in addition to increasing the expression of inflammatory cytokines [34]. The more severe lung lesions in mice infected with the HY041^T^ strain resulted from a heightened inflammatory response and the increased recruitment of inflammatory cells to the pulmonary lesions, contributing to the higher mortality of these mice. Altogether, these findings established that the HY041^T^ strain of *A*. *raozihei* exhibits enhanced pathogenicity.

In this study, we only conducted a comparative virulence analysis of *A. raozihei* strains HY037 and HY041^T^, which exhibited the most divergent pathogenicity at the survival analysis of mice, and analyzed the factors underlying their pathogenicity differences. However, survival analysis of infected mice showed that the other six strains also exhibited varying lethality rates (ranging from 40% to 70%). In the furture study we will displayed the pan-genome analysis of the eight strains and the related wet experiments to investigate the precise contributions to pathogenicity differences of these strains.

### Virulence factors of *A. raozihei*

The genetic factors underlying the pathogenesis of *A*. *raozihei* and the enhanced virulence of the HY041^T^ strain were identified through an integrated analysis combining comparative genomics and transcriptomic profiling. Comparative genomic analysis revealed that the genome sequence of *A*. *raozihei* shares high sequence similarities with 37 predicted virulence factors of *El*. *anophelis* and 26 additional virulence factors experimentally identified in other pathogens in the *Weeksellaceae* family (Table S3 and S4). Among the identified virulence factors, five genes, namely, *groL* (*HY041_GM000957*), *dnaK* (*HY041_GM002895*), *ompA* (*HY041_GM000853*), *algW* (*HY041_GM002298*), and *clpP* (*HY041_GM002093*), were found to be significantly upregulated in both the HY037 and HY041^T^ strains following cellular interactions. The present research further indicated that the expression levels of two proteases, AlgW and ClpP [35], as well as other HSP components, including DnaK, DnaJ, GrpE, GroEL, and GroES, were upregulated under high temperatures and oxidative stress [36–39] and enhancing pathogen survival. However, the bacterial HSPs encoded by the *groL* and *dnaK* genes function as antigens that enhance the humoral and cellular immune responses and are highly conserved among pathogenic species [40]. The *groL* gene of *Helicobacter pylori* induces the expression of IL-6 mRNA and the activation of NF-κB in RAW 264.7 macrophages [41]. Purified mycobacterial HSPs have been shown to trigger the dose-dependent activation of NF-κB in human endothelial cells through TLR-4 signaling [42]. According to these findings, these virulence factors may also function as antigens of *A*. *raozihei*, which contribute to the intensification of inflammatory responses in infected mice. The *ompA* gene was also upregulated in both the HY037 and HY041^T^ strains following cellular interactions and was found to share high sequence similarity with the *ompA* virulence factor of *R*. *anatipestifer*. The OmpA protein has been shown to facilitate the invasion of *R*. *anatipestifer* into brain microvascular endothelial cells and the penetration of the blood–brain barrier [43]. In our study, we also isolated *A. raozihei* strain from the brain of infected mice (Fig. S1). Therefore, we speculated that, the OmpA protein of *A. raozihei* may facilitate the invasion into the brain by increasing the adhesion and invasion capacities. The gene encoding peptidase M48 (*HY041_GM002489*) was also upregulated, and subsequent prediction of its signal peptide revealed that the protease is secretory in nature. Peptidase M48C is a member of the peptidase M48 family and functions as a mitochondrial metalloendopeptidase. Secreted proteases and metalloproteases represent crucial virulence factors in various pathogens within the *Weeksellaceae* family, including *R*. *anatipestifer* [44], *Ornithobacterium rhinotracheale*, and *C*. *indologenes* [45,46], which suggests their role as common virulence factors. These findings indicate that OmpA and peptidase M48 May serve as invasive virulence factors during *A*. *raozihei* infections.

The genes related to iron acquisition, including those encoding the TonB-dependent receptor, Fe^3+^-siderophore ABC transporter permease, and the ferric uptake regulator proteins, were found to be upregulated in both the HY037 and HY041^T^ strains following interactions with A549 cells. Iron acquisition represents a critical determinant of virulence in bacterial pathogenesis. The ability of pathogenic bacteria to obtain iron from their host is a key determinant of virulence. Conversely, to protect against invading pathogens that steal iron, hosts sequester iron using host iron-binding proteins. To overcome host iron withholding defenses, most bacterial pathogens have evolved highly sophisticated systems to acquire iron for successful infection [47]. Previous studies have demonstrated that defects in the outer membrane heme receptor gene, as well as in *tonB1* and *tonB2*, result in impaired iron uptake and slower growth, while animal experiments have demonstrated an increased median lethal dose and reduced bacterial load in the blood, liver, and brain tissues of infected ducks [47]. Yersiniabactin-like siderophore-mediated iron acquisition and heme utilization are essential for the stress adaptation and virulence of *El*. *anophelis* [48]. A previous study reported that the mild pathological damage observed in *fur* knockout strains demonstrate that it functions as a virulence factor in *R*. *anatipestifer* [25]. Therefore, Iron acquisition related genes (the genes of the TonB-dependent receptor, Fe3±siderophore ABC transporter permease) in the strains of *A.raozihei* maybe function as virulence factors and help *A.raozihei* overcoming host iron withholding defenses and acquire iron for successfully building the infection in the host.

Comparative analysis of the pathogenicity of the different strains of *A*. *raozihei* revealed significant differences in the pro-inflammatory secretory potential between the HY037 and HY041^T^ strains. The molecular mechanism underlying the enhanced pro-inflammatory potential of the HY041^T^ strain was investigated by comparative genomic analysis of the HY037 and HY041^T^ strains. The findings revealed that a few genes were differentially existence between the two strains; however, the HY041^T^ strain possessed four additional genes that were subsequently annotated as “T9SS type A sorting domain-containing protein” and “SprB protein,” suggesting that the enhanced pro-inflammatory potential is likely associated with the T9SS. Previous studies have demonstrated that T9SS is a genomic characteristic of the phylum *Bacteroidetes* [49] and serves as a virulence factor in pathogens within this phylum. The effector proteins in the T9SS of *R*. *anatipestifer*, including subtilisin-like serine protease (SspA) [50], extracellular gelatinase [51], and metallophosphoesterase [44], are members of the C10 family of serine peptidases and phosphatases that function as virulence factors and are involved in the degradation of gelatin and fibrinogen as well as the evasion of host immune surveillance. Additionally, gingipains are cysteine peptidases that function as key virulence factors and are secreted by the T9SS of *Porphyromonas gingivalis* [52]. The present study predicted that *A*. *raozihei* possesses complete structural and T9SS effector proteins. Similar to the T9SS-related virulence factors of *R*. *anatipestifer* and *Po*. *gingivalis*, three functional categories of putative T9SS effector proteins were identified by genomic analysis in this study. These predicted effector proteins were annotated as gene products with serine endopeptidase, zinc-dependent metalloprotease, and phosphatase activities, which maybe also join the degradation of cellular componets as well as the evasion of host immune surveillance, and successfully building the infection in the host.

We further speculated that the attenuated virulence of the HY037 strain is likely attributed to the absence of the *sprB* gene, which is present in the hypervirulent HY041^T^ strain. This genomic difference between the strains is significant because *sprB* partakes in biofilm formation, adhesion, and the gliding ability of *F*. *johnsoniae*. Additionally, the absence of the *sprB* gene may result in the downregulation of other proteins secreted via the T9SS and consequently attenuate the virulence of the HY037 strain. This is attributable to the co-expression of the SprB protein with the outer membrane protein, SprF, as well as the regulatory influence of SprB expression on the expression of SprC and SprD [23,53]. Our RNA-seq data revealed that the expression levels of the predicted T9SS effector genes (*HY041_GM000182* and *HY041_GM001990*) were reduced in the HY037 strain compared to those in HY041^T^, potentially as a consequence of the absence of *sprB* in HY037. Based on our experimental findings and a comprehensive review of literature, we propose that the attenuated virulence of the HY037 strain results from the absence of T9SS-related proteins, which results in the diminished expression of T9SS effectors in this strain.

Comparative transcriptomic analysis revealed that in addition to the T9SS-related proteins, the genes involved in LPS biosynthesis were also significantly differentially expressed between HY041^T^ and HY037. LPS is a well-characterized virulence factor in Gram-negative bacteria, known to activate robust inflammatory responses through TLR-4 signaling, which potentially leads to septic shock [54]. Previous study has found that LPS is recognized by Toll-like receptor 4 (TLR-4), a receptor protein on the surface of immune cells, and initiating the immune response. Once LPS binds to TLR-4, it triggers a cascade of molecules including adapter molecules like MyD88, IRAK, TRAF6, and IKKs, ultimately activate the transcription factor NF-κB. Activated NF-κB enters the nucleus and promotes the transcription of genes encoding inflammatory cytokines (like TNF-α, IL-1β, and IL-6) [54]. In our study, we found that both TNF-α and IL-6 were highly expressed in the infected mice, therefore LPS of *A. raozihei* is the critical trigger of inflammatory response through the MyD88-depending signaling pathway and causing the mice death.

Notably, A recent study demonstrated that the production of LPS in *P*. *gingivalis* is modulated by T9SS effectors, as certain effector proteins (such as RgpA, Kgp, RgpB, Hbp35, PPAD, TapA, Cpg70, PepK, and PorA) can anchor to the bacterial surface and bind A-LPS covalently [55]. We further found that HY037 strain was absent of the *sprB* gene, which was homologous to the T9SS effector of *F. johnsoniae*, the absence of the *sprB* gene may result in the downregulation of LPS biosynthesis as the decreased LPS binding sites on the cell surface of HY037 strain. Our comparative analysis revealed the concurrent differential expression of T9SS-related proteins and LPS-related genes, which suggests that variations in the expression of T9SS-related proteins may lead to differences in LPS biosynthesis and transport between HY041^T^ and HY037.

In conclusion, our study systematically investigated the pathogenicity of *A*. *raozihei* and identified its virulence factors. These findings significantly advance our understanding of the mechanisms underlying the pathogenicity of *A*. *raozihei*. Additionally, the findings provide critical insights for the development of targeted therapeutic strategies against this emerging pathogen.

## Supplementary Material

supplementary figure.docx

The ARRIVE guidelines Author Checklist.pdf

suppementary table1234589.docx

virulence_TableS67.xlsx

## Data Availability

The raw transcriptome sequence data have been deposited in the National Center for Biotechnology Information (NCBI) Sequence Read Archive (https://www.ncbi. nlm.nih.gov/sra) under accession numbers SRR22696154 ~ SRR22696156, SRR22696158 ~ SRR22696163, SRR22699715 ~ SRR22699723. The original data that support the findings of this study are openly available in (https://doi.org/10.6084/m9.figshare.29266826.v1).
