# Supplementary material for: Characterization of the pathogenicity and mechanisms underlying the pathogenesis of Apibacter raozihei, a potential bacterial pathogen
Source: Virulence. 2025 Nov 22;16(1):2586201. doi: 10.1080/21505594.2025.2586201 (PMC12645872; doi:10.1080/21505594.2025.2586201)
Supplement: supplementary figure.docx [file KVIR_A_2586201_SM4288.docx]

**Characterization of the pathogenicity and mechanisms underlying the pathogenesis of *Apibacter raozihei*, a potential bacterial pathogen**

Yuanmeihui Tao^1^, Suping Zhang^2^, Kexin Qi^1^, Wenbo Luo^1^, Sihui Zhang^3^, Jing Yang^1,4^, Dong Jin^1,4^, Shan Lu^1,4^, Yuyuan Huang^5^, Han Zheng^1,4 *^ and Jianguo Xu^1,4*^

1. National Key Laboratory of Intelligent Tracking and Forecasting for Infectious Diseases, National Institute for Communicable Disease Control and Prevention, Chinese Center for Disease Control and Prevention, Beijing, China;
2. Sichuan Center for Disease Control and Prevention, Sichuan, Chengdu, PR China;
3. Department of Epidemiology and Biostatistics, School of Public Health, Peking University, Beijing, China;

4. Research Units of Discovery of Unknown Bacteria and Function, Chinese Academy of Medical Sciences, Beijing, China;

5. Guangxi Colleges and Universities Key Laboratory of Prevention and Control of Highly Prevalent Diseases, School of Public Health, Guangxi Medical University, Nanning, Guangxi, China.

***Correspondence:** Jianguo Xu, [xujianguo@icdc.cn](mailto:xujianguo@icdc.cn). Han Zheng, [zhenghan@icdc.cn](mailto:zhenghan@icdc.cn).

**(A)**

**
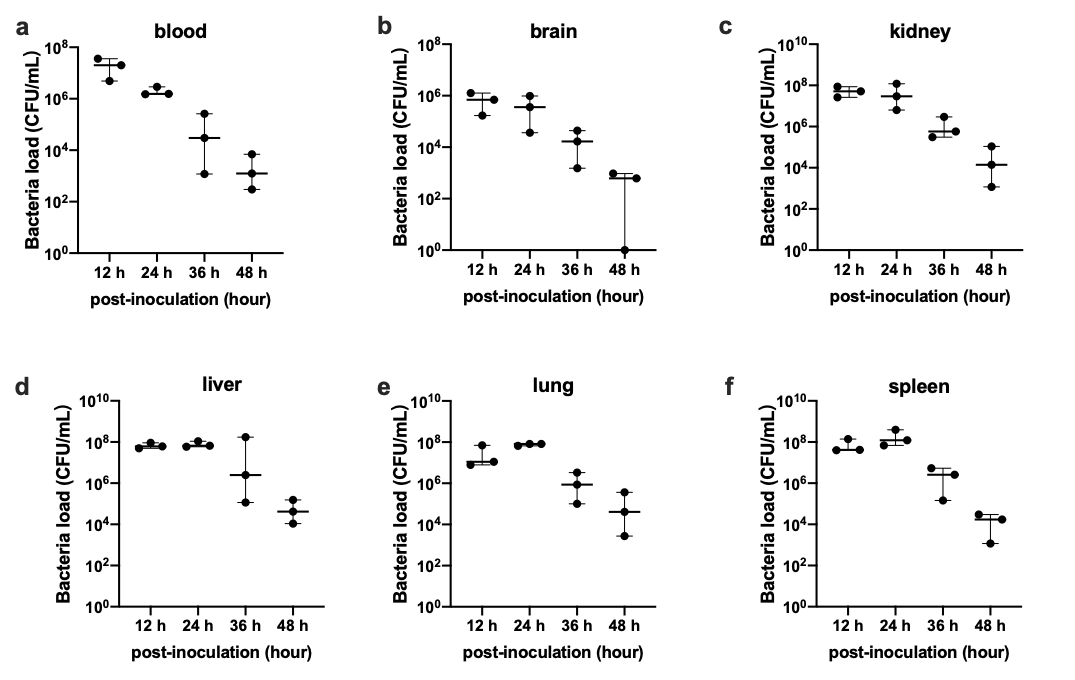
**

**(B)**


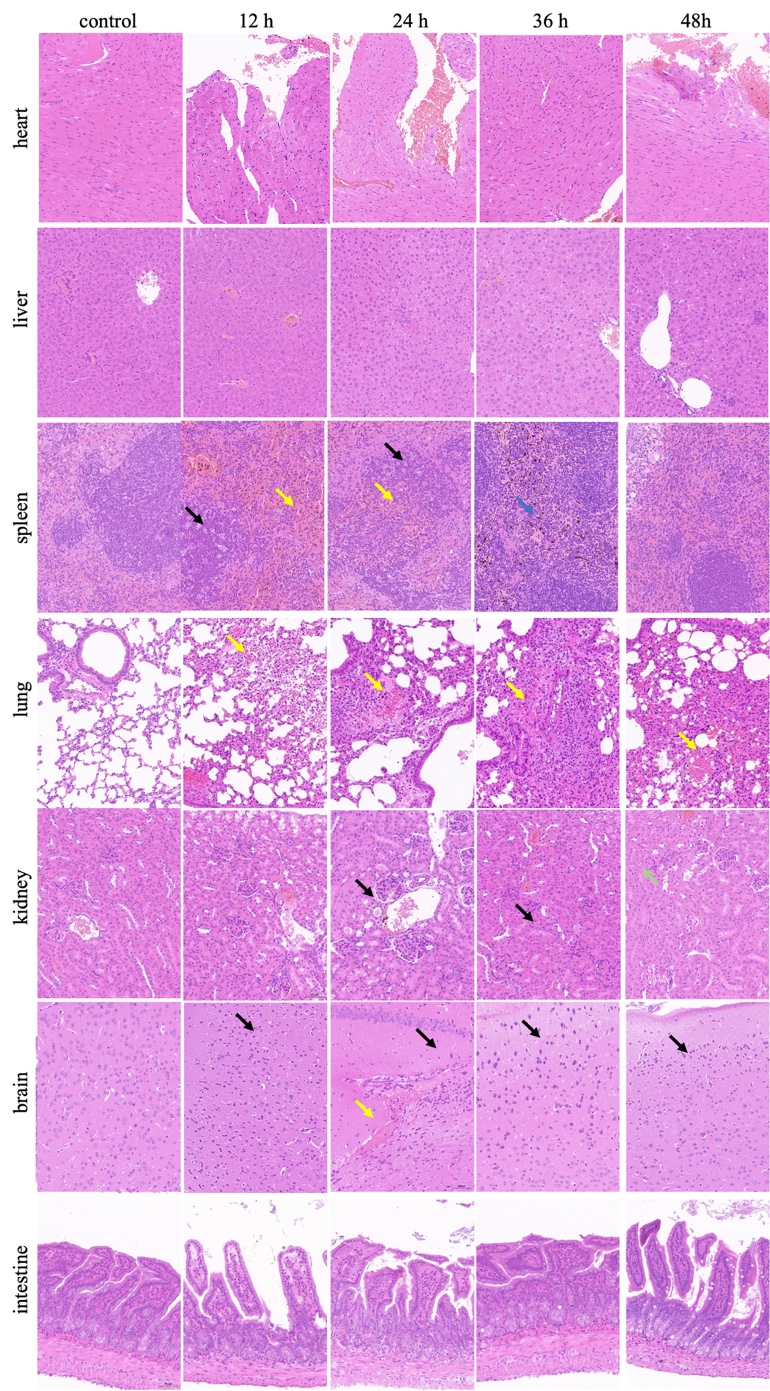


**Fig. S1. C57BL/6 mice infected by *Apibacter raozihei* strain HY041^T^**

**A:** The bacteria load in mice peripheral blood (a), brain (b), kidney (c), liver (d), lung (e), spleen (f). (C57BL/6 mice were inoculated with 10^8^ CFU per mice, Median with interquartile range, n =3, infection time: 12~48 hours).

**B:** The histology changes in C57BL/6 mice infected by *Apibacter raozihei* (C57BL/6 Mice were inoculated with 10^8^ CFU per mice, n=4, the histology changes showed in picture were as the representative. 20.0 x microscope).


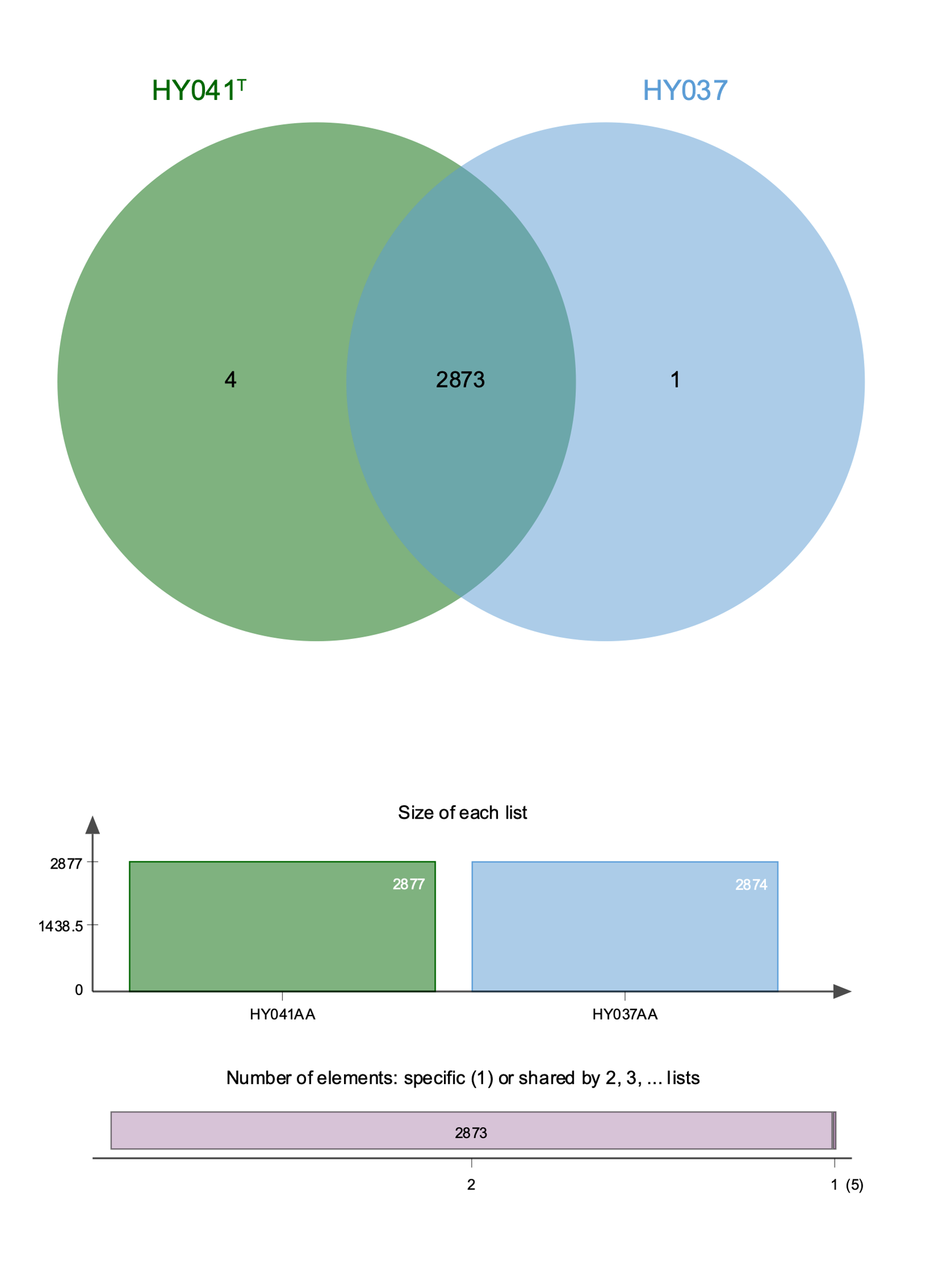


**Fig. S2. The orthologous cluster genes of strains HY041^T^ and HY037.** The comparative genomics analysis was analyzed by web server Orthovenn3 based on the amino acid sequence of bacteria.


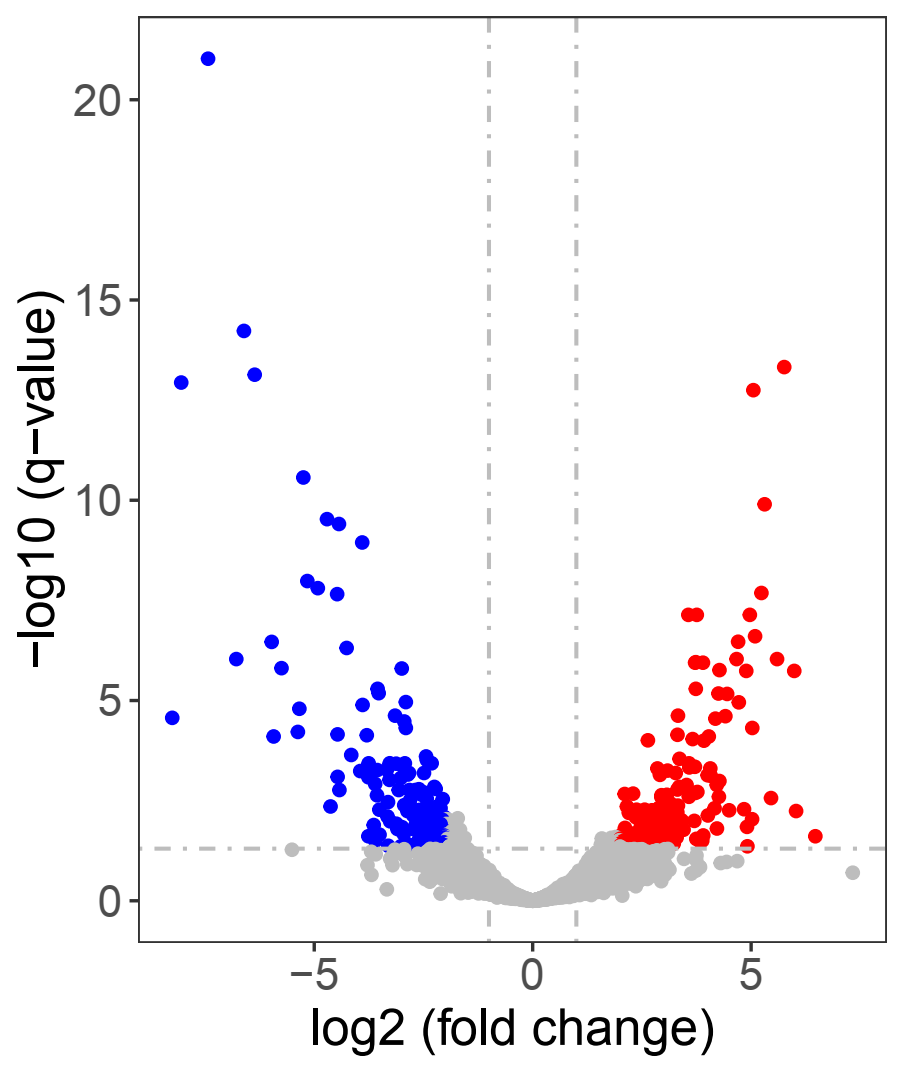

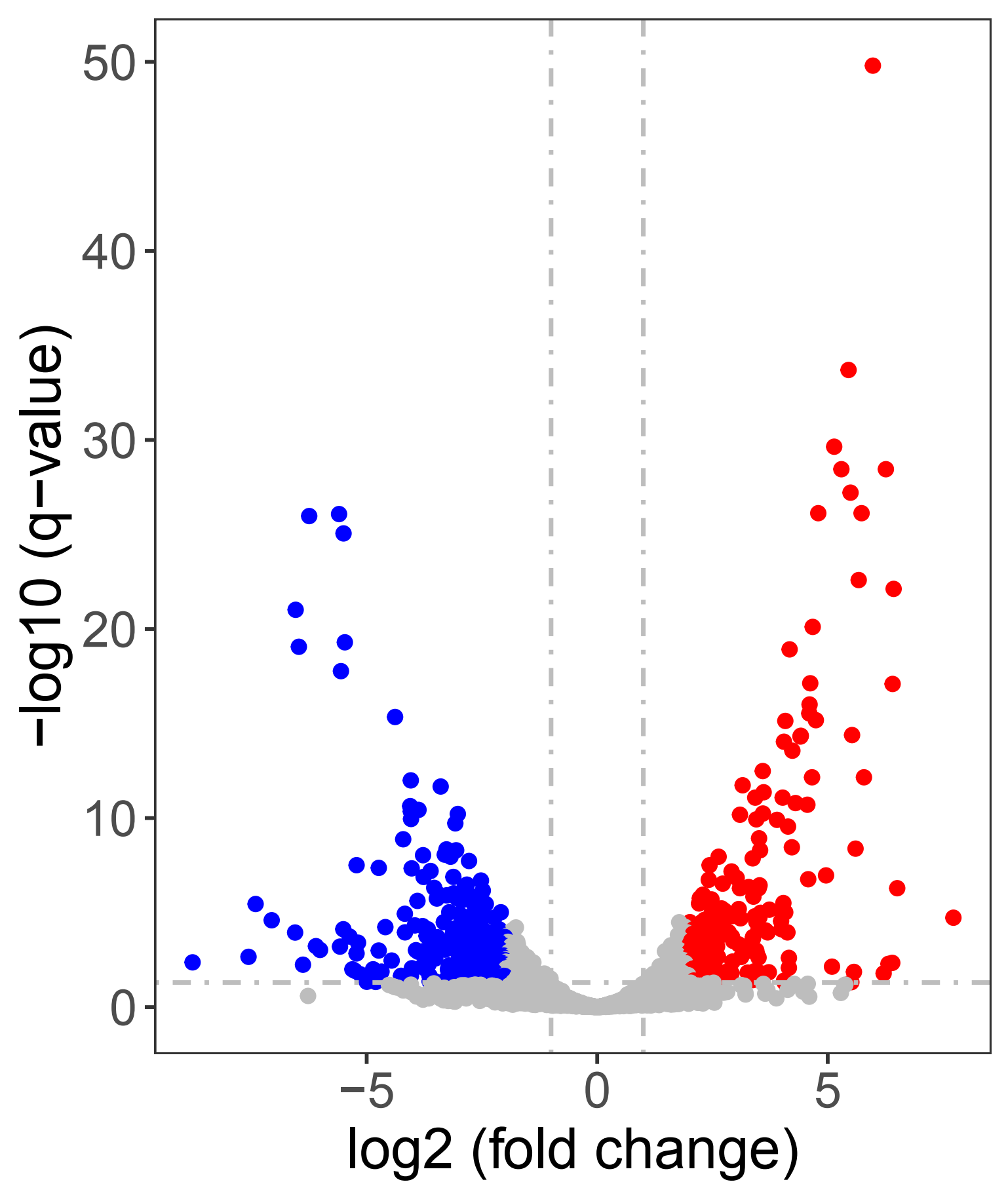

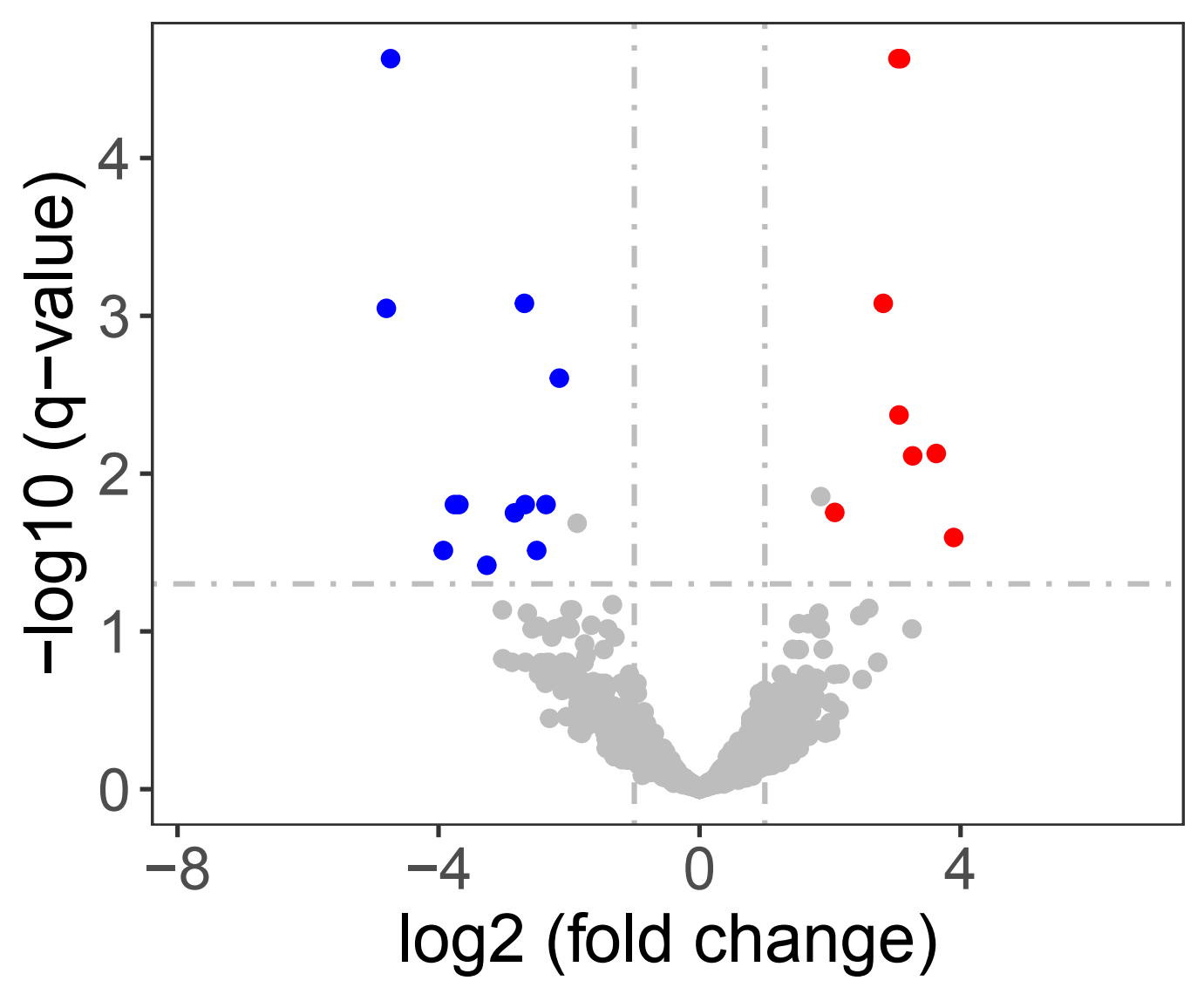

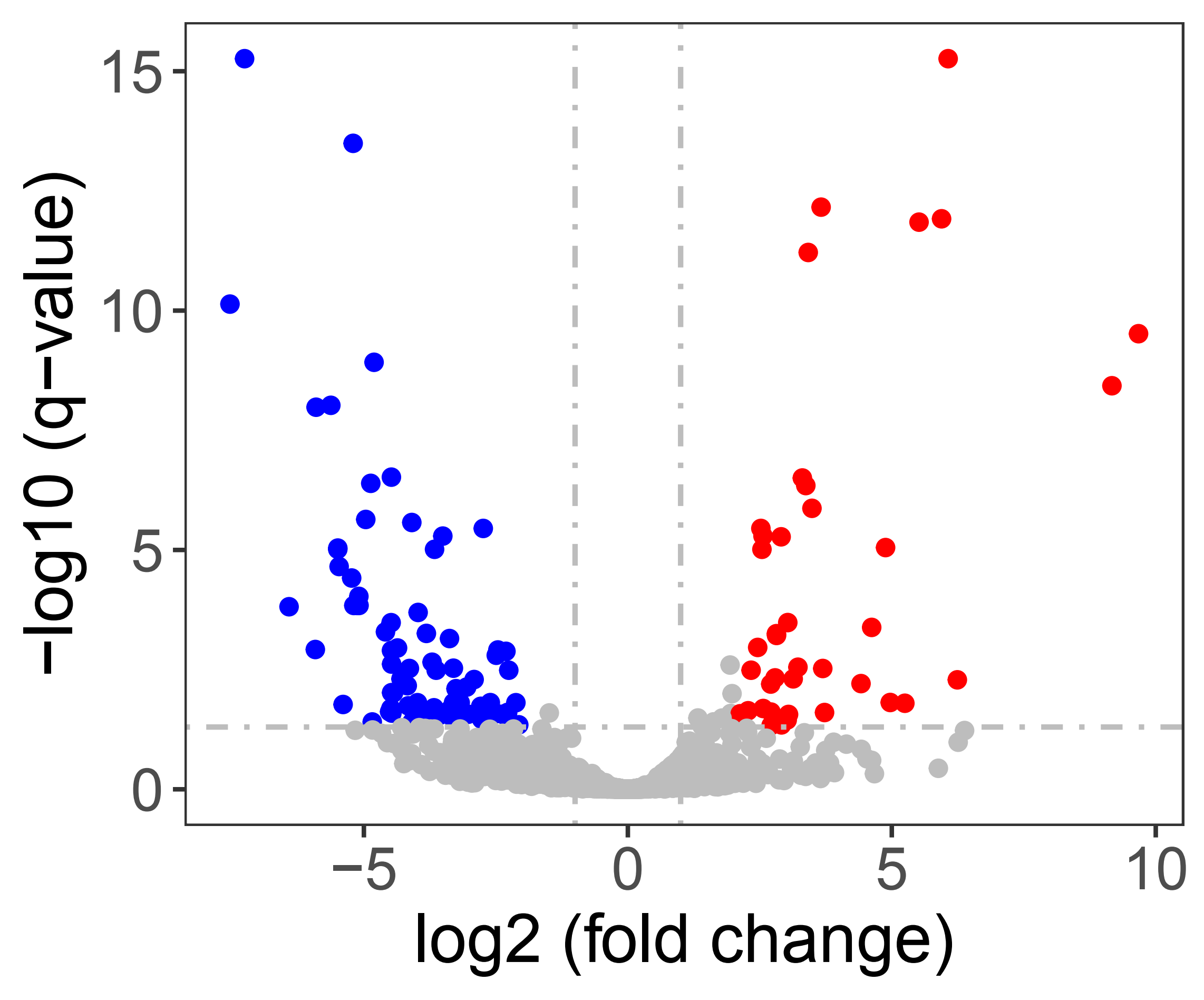

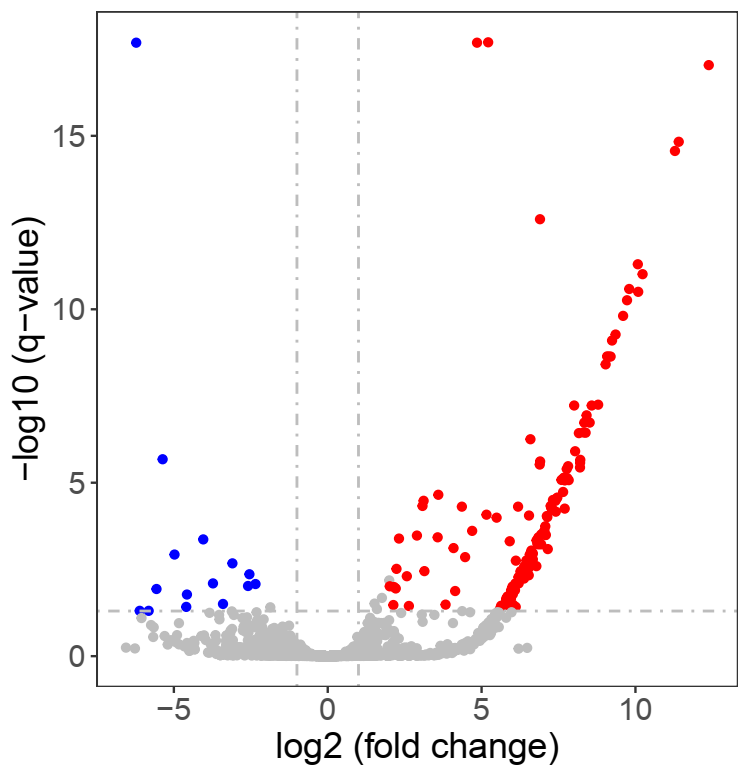

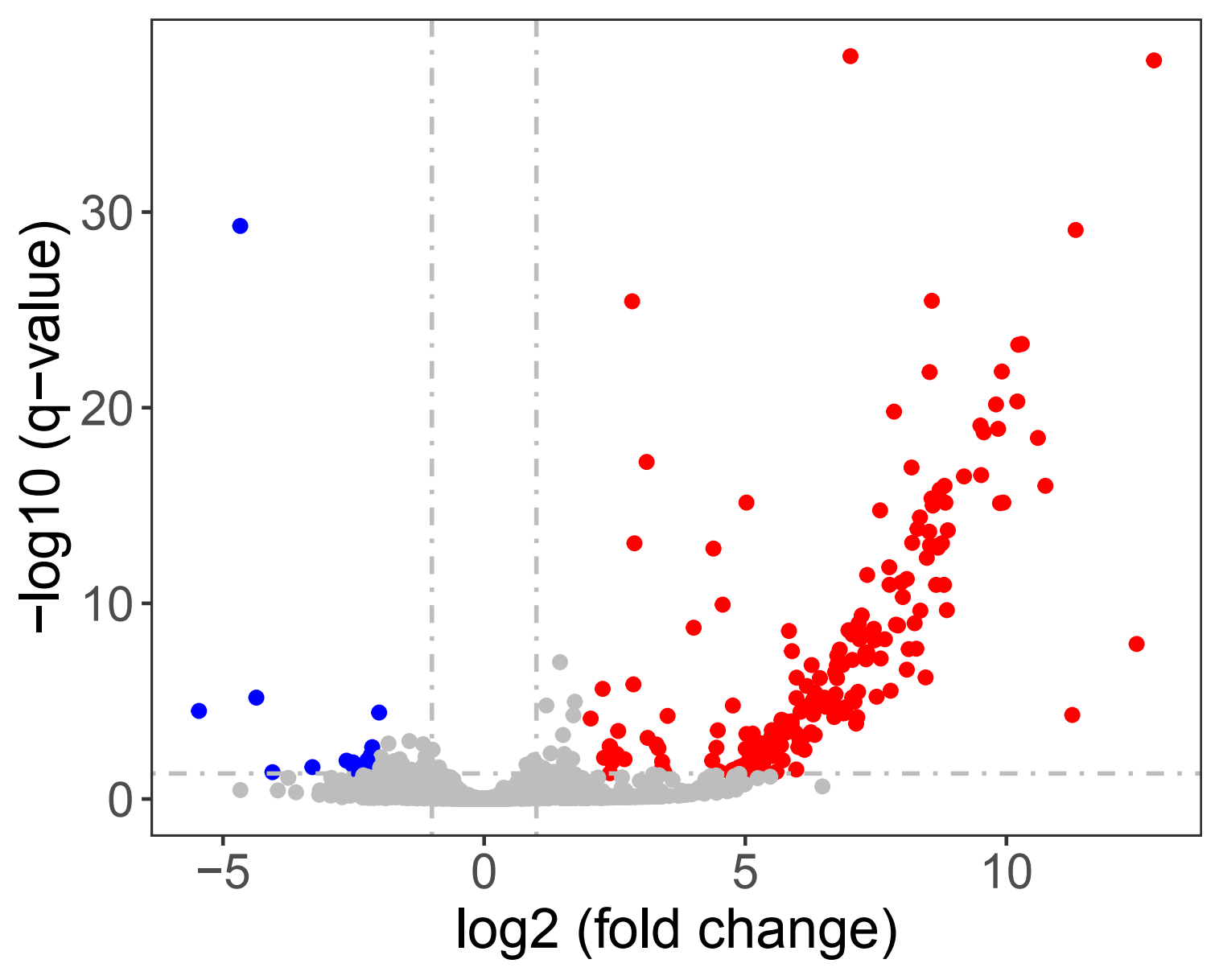


**A**

**B**

**C**

**D**

**F**

**E**

**Fig. S3. Differential expressed genes in the HY041^T^ and HY037 strains following interactions with A549 cells.** A–D: Statistical significance and fold change in the expression of DEGs of *A*. *raozihei* following interactions with A549 cells. Distribution of the DEGs in (A): HY037 and (B): HY041^T^ after 8 hours of interaction with A549 cells. Distribution of DEGs in (C): HY037 and (D) HY041^T^ after 16 hours of interaction with A549 cells. E-F: Distribution of DEGs between HY037 and HY041^T^ after 8 hours (E) and 16 hours (F) of interaction with A549 cells. The DEGs were analyzed using DESeq2, with a log_2_(fold change) cutoff of ±1.5 (n = 3). The groups that were not treated with A549 cells served as the controls for each strain.

**Table S1**. The primers for the cytokine mRNA detection and transcriptional identification. The primers were designed by NCBI-Primer website service (https://www.ncbi.nlm.nih.gov/tools/primer-blast/index.cgi?LINK_LOC=BlastHome).

**Table S2. The volume and value of RNA-seq raw data.** Three biological replicates in one group, the bacterial treatment without cells was used as the control group.

**Table S3. The summarized virulence factors of pathogens in family *Weeksellaceae* in the literature.** The virulence factors were identified by the experiment in the literature. The sequence similarities between *A. raozihei* and these factors were compared by Blast program.

**Table S4. The virulent factors of *A. raozihei* and *E. anophelis* predicted by VFDB method.** Strains of *E. anophelis* including 12012‐2 PRCM and CSID_3015183678 were the clinical strain and the outbreak strain in Wisconsin outbreak of 2016.

**Table S5. The specific gene clusters in strains HY041^T^ and HY037 genomes.** The comparative genomics analysis was analyzed by web server Orthovenn3 based on the amino acid sequence of bacteria.

**Table S6. The simultaneously upregulated homologous genes of the HY037 and HY041^T^ strains after interacting with A549 at the 8 h and 16 h.** The DEGs were analyzed using DESeq2, with a log_2_(fold change) cutoff of ±1.5 (n = 3).

**table S7. the different expressed genes between strain HY037 and HY041T after interaction with A549 cells at the 8 h and 16 h.** Differential expression genes were analyzed by DEseq2, using a log2fold change of ±1.5, n=3. No A549 cell treatment groups of each strain were used as control.

**Table S8. The predicted secretory effectors of Type IX secretion system in strains of *A. raozihei***. The effectors secreted by the Type IX secretion system was predicted by the NCBI website service CD-search based on the amino acid sequence of strain HY041^T^ and HY037 (<https://www.ncbi.nlm.nih.gov/Structure/cdd/wrpsb.cgi>).

**Table S9. The strain HY041^T^ expressed higher RHS repeat-associated core domain proteins and Rhs family proteins related genes at 8 and 16 h compared to those of HY037.** Differential expression genes were analyzed by DEseq2, using a log2fold change of ±1.5, n=3. No A549 cell treatment groups of each strain were used as control.
