# Supplementary material for: Characterization of the pathogenicity and mechanisms underlying the pathogenesis of Apibacter raozihei, a potential bacterial pathogen
Source: Virulence. 2025 Nov 22;16(1):2586201. doi: 10.1080/21505594.2025.2586201 (PMC12645872; doi:10.1080/21505594.2025.2586201)
Supplement: suppementary table1234589.docx [file KVIR_A_2586201_SM4286.docx]

| **Name** | **Amplicon size** | **Forward** | **Reverse** |
| --- | --- | --- | --- |
| **B2m** | 110 bp | ATGGCTCGCTCGGTGACCCT | TTCTCCGGTGGGTGGCGTGA |
| **TNF-α** | 257 bp | ATGGCCTCCCTCTCATCAGTT | ACAGGCTTGTCACTCGAATTTTG |
| **IL-6** | 134 bp | CTTCCATCCAGTTGCCTTCT | CTCCGACTTGTGAAGTGGTATAG |
| **MCP-1** | 101 bp | CTCACCTGCTGCTACTCATTC | ACTACAGCTTCTTTGGGACAC |
| **β2m** | 111 bp | GAACCAGCAGTTCCCTACCC | CTACCTGGCATGCCCTTTGA |
| **HY041_GM001299** | 92 bp | CGCAAAGGATGTCCTAACCA | ACAACAATCGGCAACTTACTGT |
| **HY041_GM001301** | 144 bp | CTGCCATTTACGTTTCCGCT | TCGCCTGTATAACTGCTGCTT |
| **HY041_GM001302** | 135 bp | AGGAATGCCGGTTGAAATGAC | AGCTCCGGATTAGCCATCAC |
| **HY041_GM001765** | 117 bp | TTCTGGTGTTTAAGTCCGTGCT | GGTTGTTCCACTGGATACCTCT |
| **HY041_GM001855** | 118 bp | GATCGGTATGTGTGGTCGCT | GCGGATGCCTCATCATGGTA |
| **HY041_GM001486** | 73 bp | GTGGTGCGGTCCCTGTAAAA | AGTGAGCTGTTCACCCAGTTT |
| **HY041_GM002799** | 190 bp | TGCCGGAAGAAGAAGCTACC | GGGGAAAAACCTATTCCCGGT |
| **HY041_GM000690** | 107 bp | TGGCCAAGTTTTGACCGAGA | TGACTACCGCAATTGGCACA |
| **HY041_GM001682** | 91 bp | TTGGCTGTGGCGGAACATTA | CCACGGGTACCAAGTTCTCC |
| **HY041_GM002272** | 140 bp | TCCGTAGGAGACAGTTCGGT | TTTCCTTCTCCTGCTACGGC |
| **HY041_GM002298** | 154bp | CCGGAACCGGAACCTTTGAA | TATTGTTTTACGGCGCTGGC |
| **HY041_GM000957** | 133 bp | GCTTGCTGCTGGGATCATTG | TGGCATCGCAGGTTCATCTT |
| **HY041_GM001135** | 135 bp | CGGGCATGGTGATCCTTCTT | CAGGGCCGGTTGTATGGTAG |
| **HY041_GM001186** | 177 bp | GCGCTTAGGGCAGTCTCTTT | AAGGCGTCCTGTTTTCCGTA |
| **HY041_GM002895** | 193 bp | CGTAGGAGCCGCTATTCAGG | TACAGCGGGCTGATTGTCTG |

**Table S1**. The primers for the cytokine mRNA detection and transcriptional identification. The primers were designed by NCBI-Primer website service (<https://www.ncbi.nlm.nih.gov/tools/primer-blast/index.cgi?LINK_LOC=BlastHome>).

**Table S2. The volume and value of RNA-seq raw data.** Three biological replicates in one group, the bacterial treatment without cells was used as the control group.

|  | sample id | total reads | Q20 percentage(%) | mRNA reads | map percentage(%) |
| --- | --- | --- | --- | --- | --- |
| control group (0 h) | 37_1 | 6.50E+07 | 98.2 | 2.07E+06 | 46.9 |
|  | 37_2 | 6.33E+07 | 96.4 | 1.79E+06 | 97.4 |
|  | 37_3 | 7.73E+07 | 98.6 | 2.02E+06 | 91.9 |
|  | 41_1 | 2.49E+07 | 98.3 | 1.90E+06 | 97.3 |
|  | 41_2 | 8.98E+07 | 90.4 | 1.79E+06 | 93.5 |
|  | 41_3 | 5.26E+07 | 98.5 | 1.73E+06 | 95.2 |
| 8 h treatment | 37_8_1 | 6.32E+07 | 98.3 | 1.69E+06 | 97.3 |
|  | 37_8_2 | 9.11E+07 | 98.7 | 2.21E+06 | 98.9 |
|  | 37_8_3 | 5.54E+07 | 98.6 | 2.12E+06 | 80.5 |
|  | 41_8_1 | 2.67E+07 | 98.6 | 1.72E+06 | 92.7 |
|  | 41_8_2 | 1.77E+08 | 98.3 | 2.72E+06 | 84.4 |
|  | 41_8_3 | 1.21E+08 | 98.3 | 1.61E+06 | 79.7 |
| 16 h treatment | 37_16_1 | 5.95E+07 | 98.6 | 1.67E+06 | 96.9 |
|  | 37_16_2 | 1.67E+07 | 98.4 | 2.90E+06 | 94.7 |
|  | 37_16_3 | 9.45E+06 | 92.1 | 2.20E+06 | 70.7 |
|  | 41_16_1 | 4.18E+07 | 98.3 | 1.90E+06 | 96.3 |
|  | 41_16_2 | 1.09E+08 | 96.7 | 1.92E+06 | 98.1 |
|  | 41_16_3 | 2.92E+07 | 93.2 | 2.47E+06 | 93.3 |

**Table S3. The summarized virulence factors of pathogens in family *Weeksellaceae* in the literature.** The virulence factors were identified by the experiment in the literature. The sequence similarities between *A. raozihei* and these factors were compared by Blast program.

| Reference (PMID) | Virulence factors |  | HY041 locus | coverage (%) | identity (%) |
| --- | --- | --- | --- | --- | --- |
| 36508857 | GldG | AS87_RS00460 | HY041_GM000055 | 95 | 49.06 |
| 31164171 | GldM | AS87_RS04190 | HY041_GM002930 | 88 | 67.16 |
| 30955831 | Gldk | AS87_RS08465 | HY041_GM002932 | - | - |
| 30640518 | sprA | AS87_RS08785 | HY041_GM002813 | 87 | 57.7 |
| 35677936 | PncA | AS87_RS01745 | HY041_GM002913 | 9 | 74.58 |
| 29312236 | C5a peptidase | AS87_RS00980 | - | - | - |
|  | S8 family serine peptidase | RAYM_01812 | - | - | - |
| 33741629 | metallophosphoesterase | RAYM_04099 | HY041_GM001093 | - | - |
| 31281307 | LptD |  | HY041_GM001881 | 89 | 49.76 |
| 27500736 | recombinase | LptD | HY041_GM002245 | 70 | 43.4 |
| 25804836 | DUF5689 domain-containing protein | M949_1360 | HY041_GM000947 | 22 | 36.96 |
| 26266750 | glycosyltransferase family 2 protein | M949_1556 | HY041_GM000793 | 41 | 39.81 |
| 30223890 | CPBP family intramembrane metalloprotease | M949_1603 | HY041_GM002648 | 22 | 46.15 |
| 28166822 | bacterial lipopolysaccharide biosynthesis proteins | M949_RS01035 | HY041_GM001726 | 22 | 33.33 |
| 25303276 | Vi polysaccharide biosynthesis protein VipB/TviC | M949_RS01915 | - | - | - |
| 26928424 | acyl transferase | AS87_04050 | HY041_GM002497 | 90 | 65.9 |
| 28442426 | wza-like gene | AS87_03730 | HY041_GM002542 | 91 | 38.91 |
| 34616377 | OmpH | WP_014938071.1 | HY041_GM000447 | 99 | 40.6 |
| 28284604 | hemin receptor | B739_0832 | HY041_GM000565 | 50 | 28.75 |
| 36863175 | ompA | B739_1208 | HY041_GM002736 | 91 | 61.74 |
|  |  | GE296_RS00400 | HY041_GM000842 | 67 | 63.22 |
|  |  | GE296_RS02455 | HY041_GM000195 | 75 | 73.5 |
|  |  | GE296_RS02910 | HY041_GM001049 | 73 | 74.67 |
|  |  | GE296_RS05220 | HY041_GM000853 | 97 | 52.26 |
| 33579370 | outer membrane transport energization protein ExbD | GE296_RS06915 | HY041_GM000845 | 85 | 43.4 |
|  | TonB-dependent receptor plug | Riean_0932 |  |  |  |
|  | riboflavin biosynthesis protein RibD | Riean_1561 | - | - | - |
| 26293113 | TonB1 and TonB2 | Riean_1661 | HY041_GM000844 | 32 | 44 |
| 28971067 | Ferric uptake regulator (Fur) | KM393215.1/ KM393216.1 | HY041_GM001766 | 93 | 57.5 |
| 11889100 | CAMP cohemolysin | RAYM_04846 | HY041_GM002487 | 98 | 66.57 |
| 22038245 | chaperonin GroEL | AF202727 | HY041_GM000957 | 99 | 85.6 |
| 8926061 | serine protease/hemagglutinin | U94318/L31763 | - |  |  |
| 29284206 | ArsR and SthK | RAYM_RS09735/RAYM_RS09740 | - | - | - |

**Table S4. The virulent factors of *A. raozihei* and *E. anophelis* predicted by VFDB method.** Strains of *E. anophelis* including 12012‐2 PRCM and CSID_3015183678 were the clinical strain and the outbreak strain in Wisconsin outbreak of 2016.

| *E. anophelis* | | *Apibacter raozihei* |  |  |
| --- | --- | --- | --- | --- |
| CSID_3015183678 | 12012‐2 PRCM | HY041 | VF_name | Related_genes |
| **-** | **+** | HY041_GM001765 | ﻿Methionine sulphoxide reductase | MsrA ﻿Peptide methionine sulfoxide reductase |
| **+** | **-** | **HY041_GM002298** | **Alginate regulation** | **algW peptidase, S1C protease Do subfamily** |
| **+** | **+** | HY041_GM000804 | Capsular polysaccharide | rmlA Glucose-1-phosphate thymidylyltransferase |
| **+** | **-** | **HY041_GM000807** | **Capsule** | **BJAB07104_00096 putative UDP-glucose 6-dehydrogenase** |
| **+** | **-** | **HY041_GM000806** | **Capsule** | **M3Q_285 nucleoside-diphosphate sugar epimerase** |
| **+** | **-** | **HY041_GM000450** | **Capsule** | **uppS undecaprenyl diphosphate synthase** |
| **+** | **-** | **HY041_GM002799** | **Catalase** | **katA catalase** |
| **+** | **+** | HY041_GM002093 | ClpP | clpP ATP-dependent Clp protease proteolytic subunit |
| **+** | **-** | **HY041_GM002000** | **colibactin** | **clbD putative 3-hydroxyacyl-CoA dehydrogenase** |
| **+** | **-** | **HY041_GM002003** | **Copper exporter** | **ctpV Putative metal cation transporter P-type ATPase CtpV** |
| **+** | **+** | HY041_GM000075 | EF-Tu | tufA elongation factor Tu |
| **+** | **+** | HY041_GM002887 | Exopolysaccharide | pgi glucose-6-phosphate isomerase |
| **+** | **-** | **HY041_GM000773** | **Heme biosynthesis** | **hemB Porphobilinogen synthase** |
| **+** | **-** | **HY041_GM000574** | **Heme biosynthesis** | **hemE uroporphyrinogen decarboxylase** |
| **+** | **+** | HY041_GM000715 | Heme biosynthesis | hemL glutamate-1-semialdehyde aminotransferase |
| **+** | **+** | HY041_GM000957 | Hsp60 | htpB Hsp60, 60K heat shock protein HtpB |
| **+** | **+** | HY041_GM000677 | IlpA | IlpA immunogenic lipoprotein A |
| **+** | - | HY041_GM001355 | Leucine synthesis | leuD 3-isopropylmalate dehydratase, small subunit |
| **+** | **-** | **HY041_GM000798** | **LOS** | **C8J_1080 hypothetical protein** |
| **+** | **+** | HY041_GM001507 | LOS | galE UDP-glucose 4-epimerase |
| **+** | **-** | **HY041_GM002263** | **LOS** | **kpsF KpsF** |
| **+** | **-** | **HY041_GM001885** | **LOS** | **orfM deoxyribonucleotide triphosphate pyrophosphatase** |
| **+** | **-** | **HY041_GM000412** | **LPS** | **hisF imidazole glycerol phosphate synthase subunit HisF** |
| **+** | **-** | **HY041_GM002200** | **LPS** | **kdsA 2-dehydro-3-deoxyphosphooctonate aldolase** |
| **+** | **-** | **HY041_GM002204** | **LPS** | **kdtB lipopolysaccharide core biosynthesis protein** |
| **+** | **-** | **HY041_GM000471** | **LPS** | **lpg0756 dTDP-6-deoxy-D-glucose-3,5-epimerase RmlC** |
| **+** | **+** | HY041_GM000328 | Mg2+ transport | mgtB magnesium-translocating P-type ATPase |
| **+** | **-** | **HY041_GM001622** | **Mip** | **mip macrophage infectivity potentiator Mip** |
| **+** | **+** | HY041_GM002895 | MOMP | CT396 molecular chaperone DnaK |
| **+** | **-** | **HY041_GM000386** | **Nucleoside diphosphate kinase** | **ndk nucleoside diphosphate kinase** |
| **+** | **-** | **HY041_GM001697** | **Pantothenate synthesis** | **panC pantoate--beta-alanine ligase PanC** |
| **+** | **+** | HY041_GM001563 | Polar flagella | flmH 3-oxoacyl-ACP reductase |
| **+** | **-** | **HY041_GM000316** | **SodB** | **sodB superoxide dismutase** |
| **+** | **-** | **HY041_GM000366** | **SodB** | **sodB superoxide dismutase** |
| **+** | **-** | **HY041_GM002134** | **SodB** | **sodB superoxide dismutase** |
| **+** | **+** | HY041_GM002923 | Streptococcal enolase | eno enolase |
| **+** | **-** | **HY041_GM001759** | **Streptococcal plasmin receptor/GAPDH** | **plr/gapA Glyceraldehyde 3-phosphate dehydrogenase, putative** |
| **+** | **-** | **HY041_GM002792** | **Type IV pili biosynthesis** | **pilR putative two-component system, response regulator** |

**Table S5. The specific gene clusters in strains HY041^T^ and HY037 genomes.** The comparative genomics analysis was analyzed by web server Orthovenn3 based on the amino acid sequence of bacteria.

|  | gene locus | PFAMs annoation |
| --- | --- | --- |
| HY041 specific gene clusters | | |
| cluster 1 | HY041_GM000292 | RHS_repeat,SpvB,TcdB_toxin_midC,TcdB_toxin_midN |
|  | HY041_GM000539 | RHS_repeat,SpvB,TcdB_toxin_midC,TcdB_toxin_midN |
| cluster 2 | HY041_GM000346 | CHU_C,PKD,SASA,SprB |
|  | HY041_GM001761 | CHU_C,PKD,SASA,SprB |
| cluster 3 | HY041_GM001607 | hypothetical protein |
|  | HY041_GM001763 | hypothetical protein |
| cluster 4 | HY041_GM000277 | hypothetical protein |
|  | HY041_GM000280 | hypothetical protein |
|  | HY041_GM000282 | hypothetical protein |
|  | HY041_GM000285 | hypothetical protein |
|  | HY041_GM000290 | hypothetical protein |
|  | HY041_GM000523 | hypothetical protein |
|  | HY041_GM000529 | hypothetical protein |
|  | HY037 specific genes cluster | |
| cluster 1 | HY037_GM002248 | hypothetical protein |
|  | HY037_GM002251 | hypothetical protein |
|  | HY037_GM002253 | hypothetical protein |
|  | HY037_GM002256 | hypothetical protein |
|  | HY037_GM002261 | hypothetical protein |
|  | HY037_GM002440 | hypothetical protein |
|  | HY037_GM002446 | hypothetical protein |

**Table S8. The predicted secretory effectors of Type IX secretion system in strains of *A. raozihei***. The effectors secreted by the Type IX secretion system was predicted by the NCBI website service CD-search based on the amino acid sequence of strain HY041^T^ and HY037 (<https://www.ncbi.nlm.nih.gov/Structure/cdd/wrpsb.cgi>).

| Gene_id | | Annoation | Secretory signal/ gene |
| --- | --- | --- | --- |
| HY041_GM000182 | HY037_GM002153 | - | YES |
| HY041_GM000243 | HY037_GM002214 | Por secretion system C-terminal sorting domain-containing protein | - |
| HY041_GM000293 | HY037_GM002954 | Por secretion system C-terminal sorting domain-containing protein [Apibacter mensalis] | YES |
| HY041_GM000540 | HY037_GM002954 | Por secretion system C-terminal sorting domain-containing protein [Apibacter mensalis] | YES |
| HY041_GM001020 | HY037_GM001091 | T9SS C-terminal target domain-containing protein [Flavobacterium sp. Leaf359] | - |
| HY041_GM001092 | HY037_GM001163 | serine-type endopeptidase activity; proteolysis | - |
| HY041_GM001093 | HY037_GM001164 | proteolysis; serine-type endopeptidase activity | - |
| HY041_GM001094 | HY037_GM001165 | hypothetical protein [Apibacter mensalis] | YES |
| HY041_GM001136 | HY037_GM001207 | Por secretion system C-terminal sorting domain-containing protein [Apibacter mensalis] | YES |
| HY041_GM001262 | HY037_GM001334 | zinc ion binding; proteolysis; metalloendopeptidase activity; | - |
| HY041_GM001263 | HY037_GM001333 | proteolysis; extracellular matrix; metalloendopeptidase activity | - |
| HY041_GM001288 | HY037_GM002375 | - | YES |
| HY041_GM001424 | HY037_GM001495 | phosphatase activity; | YES |
| HY041_GM001672 | HY037_GM002721 | cysteine-type peptidase activity; acid phosphatase activity;proteolysis; metal ion binding; | YES |
| HY041_GM001673 | HY037_GM002722 | serine-type endopeptidase activity; metal ion binding; acid phosphatase activity; proteolysis; | - |
| HY041_GM001675 | HY037_GM002717 | DNA ligase (ATP) activity; DNA recombination; | - |
| HY041_GM001713 | HY037_GM002680 | - | - |
| HY041_GM001761 |  | sprB; photosynthesis; outer membrane; | - |
| HY041_GM001959 | HY037_GM000195 | type IX secretion system membrane protein, PorP/SprF family [Apibacter mensalis] | YES |
| HY041_GM001988 | HY037_GM000227 | calcium ion binding; | - |
| HY041_GM001990 | HY037_GM000226 | calcium ion binding; | YES |
| HY041_GM001991 | HY037_GM000225 | calcium ion binding; | YES |
| HY041_GM002303 | HY037_GM000539 | gliding motility-associated C-terminal domain-containing protein [Apibacter mensalis] | - |
| HY041_GM002388 | HY037_GM001997 | proteolysis; cysteine-type peptidase activity; | YES |
| HY041_GM002967 | HY037_GM001967 | - | - |
| HY041_GM000012 | HY037_GM001983 | gliding motility-associated protein GldE | *gldE* |
| HY041_GM000013 | HY037_GM001984 | Gliding motility protein GldD | *gldD* |
| HY041_GM000055 | HY037_GM002026 | ABC-type uncharacterized transport system | *gldG* |
| HY041_GM000056 | HY037_GM002027 | Gliding motility protein Gldf | *gldF* |
| HY041_GM000583 | HY037_GM002494 | Gliding motility-associated lipoprotein, GldH | *gldH* |
| HY041_GM000585 | HY037_GM002496 | SprT-like family | *sprT* |
| HY041_GM000774 | HY037_GM000845 | Gliding motility protein GldC | *gldC* |
| HY041_GM000775 | HY037_GM000846 | Gliding motility protein, GldB | *gldB* |
| HY041_GM001812 | HY037_GM000049 | long-chain fatty acid transport protein | *porV* |
| HY041_GM001813 | HY037_GM000050 | Peptidase family C25 | *porU* |
| HY041_GM001959 | HY037_GM000195 | Type IX secretion system membrane protein PorP/SprF | *-* |
| HY041_GM002813 | HY037_GM001813 | SprA protein | *sprA* |
| HY041_GM002929 | HY037_GM001929 | gliding motility GldN | *gldN* |
| HY041_GM002930 | HY037_GM001930 | Gliding motility protein GldM | *gldM* |
| HY041_GM002931 | HY037_GM001931 | Gliding motility protein GldL | *gldL* |
| HY041_GM002932 | HY037_GM001932 | Gliding motility protein Gldk | *gldK* |
| HY041_GM002172 | HY037_GM000408 | Chemotaxis protein CheY | *porX* |
| HY041_GM001598 | HY037_GM002869 | Histidine kinase | *porY* |

**Table S9. The strain HY041^T^ expressed higher RHS repeat-associated core domain proteins and Rhs family proteins related genes at 8 and 16 h compared to those of HY037.** Differential expression genes were analyzed by DEseq2, using a log2fold change of ±1.5, n=3. No A549 cell treatment groups of each strain were used as control.

| HY041_gene locus | log2FoldChange | | eggNOG annoation |
| --- | --- | --- | --- |
|  | 8h | 16h |  |
| **HY041_GM000182** | **0.3** | **1.5** | **RHS repeat-associated core domain protein** |
| HY041_GM000242 | 3.1 | 5.2 | RHS repeat-associated core domain protein |
| **HY041_GM000243** | **5.4** | **5.1** | **Por secretion system C-terminal sorting domain-containing protein** |
| HY041_GM000279 | 8.8 | 6.1 | RHS repeat-associated core domain protein |
| HY041_GM000281 | 6.9 | 5.8 | RHS repeat-associated core domain |
| HY041_GM000284 | 6.3 | 6.4 | RHS repeat-associated core domain protein |
| HY041_GM000287 | 8.9 | 9.0 | RHS repeat-associated core domain protein |
| HY041_GM000292 | 0.7 | 2.6 | RHS repeat-associated core domain protein |
| HY041_GM000997 | 5.0 | 4.5 | RHS repeat-associated core domain |
| HY041_GM000999 | 6.9 | 1.9 | COG3209 Rhs family protein(rhsC) |
| HY041_GM001003 | 7.0 | 4.6 | RHS repeat-associated core domain |
| HY041_GM001013 | 6.7 | 5.6 | RHS repeat-associated core domain |
| HY041_GM001017 | 3.5 | 3.6 | RHS repeat-associated core domain |
| HY041_GM001019 | 7.3 | 7.6 | TIGRFAM YD repeat |
| HY041_GM001911 | 7.5 | 6.1 | RHS repeat-associated core domain protein |
| HY041_GM001914 | 7.8 | 6.6 | RHS repeat-associated core domain protein |
| HY041_GM001917 | 7.5 | 7.4 | COG3209 Rhs family protein |
| HY041_GM001919 | 7.8 | 7.7 | TIGRFAM YD repeat protein |
| HY041_GM001921 | 12.8 | 11.3 | type IV secretion protein Rhs |
| **HY041_GM001990** | **6.7** | **6.6** | **Por secretion system C-terminal sorting domain-containing protein** |
| HY041_GM002140 | 9.9 | 9.6 | COG3209 Rhs family protein(rhsA) |
| HY041_GM002143 | 9.9 | 8.6 | COG3209 Rhs family protein |
| HY041_GM002145 | 4.7 | 6.8 | COG3209 Rhs family protein |
| HY041_GM002152 | 5.7 | 5.5 | COG3209 Rhs family protein |
| HY041_GM002153 | 8.3 | 6.8 | TIGRFAM RHS repeat-associated core |
| HY041_GM002580 | 10.7 | 9.1 | COG3209 Rhs family protein |
| HY041_GM001630 | 8.8 | 6.0 | COG3209 Rhs family protein |
